# Discovery of new genetic loci for male sexual orientation in Han population

**DOI:** 10.1038/s41421-021-00341-7

**Published:** 2021-10-31

**Authors:** Shao-Hua Hu, Hai-mei Li, Hao Yu, Yan Liu, Chen-Xing Liu, Xian-bo Zuo, Jing Lu, Jia-Jun Jiang, Cai-Xi Xi, Bo-Chao Huang, Hu-Ji Xu, Jian-Bo Hu, Jian-Bo Lai, Man-Li Huang, Jian-Ning Liu, Dan-Ge Xu, Xi-Chao Guo, Wei Wu, Xin Wu, Lei Jiang, Meng Li, Guang-Ping Zhang, Jin-Wen Huang, Ning Wei, Wen Lv, Jin-Feng Duan, Hong-Li Qi, Chan-Chan Hu, Jing-Kai Chen, Wei-Hua Zhou, Wei-Juan Xu, Chen-Feng Liu, Hai-Yong Liang, Jing Du, Shu-Fa Zheng, Qiao-Ling Lu, Lin Zheng, Xiao-Wei Hu, Feng-Xiang Chen, Peng Chen, Biao Zhu, Li-Jun Xu, Zhi-Min Ni, Ye-Zhen Fang, Zuo-Kai Yang, Xin-Ren Shan, En-de Zheng, Fan Zhang, Qing-qing Zhou, Yi Rao, Dick Swaab, Wei-Hua Yue, Yi Xu

**Affiliations:** 1 Department of Psychiatry, First Affiliated Hospital, Zhejiang University School of Medicine, Hangzhou, Zhejiang, China; 2 The Key Laboratory of Mental Disorder Management in Zhejiang Province, Hangzhou, Zhejiang, China; 3 National Human Brain Bank for Health and Disease, Hangzhou, Hangzhou, Zhejiang, China; 4 Brain Research Institute, Zhejiang University, Hangzhou, Zhejiang, China; 5 Department of Psychiatry, Jining Medical University, 133 Hehua Rd, Jining, Shandong China; 6 Department of Neurobiology, School of Basic Medical Sciences, Capital Medical University, Beijing, China; 7 Department of Human Genetics, University of Pittsburgh, Pittsburgh, PA USA; 8 Institute of Dermatology and Department of Dermatology at First Hospital, Anhui Medical University, Hefei, Anhui China; 9 Department of General Office, Center for Disease Control of Jianggan District, Hangzhou, Zhejiang, China; 10 Department of Clinical Laboratory, First Affiliated Hospital, Zhejiang University School of Medicine, Hangzhou, Zhejiang, China; 11 State Key Laboratory for Diagnosis and Treatment of Infectious Disease, First Affiliated Hospital, Zhejiang University School of Medicine, Hangzhou, Zhejiang, China; 12 Department of Rheumatology and Immunology, Shanghai Changzheng Hospital, The Second Military Medial University, Shanghai, China; 13 Department of Psychiatry, Seventh Hangzhou Hospital, Hangzhou, Zhejiang, China; 14 Medicines&Biochemical Products Branch, Zhejiang Medicine &Health Products I/E CO., Ltd, Hangzhou, Zhejiang, China; 15 BioMiao Biological Technology (Beijing) Co., Ltd, Beijing, China; 16 Beijing Emei Tongde Technology Development Co., Ltd, Beijing, China; 17 Department of Aids Venereal Disease Control and Prevention, Shaoxing Center for Disease Control and Prevention, Shaoxing, Zhejiang, China; 18 Department of Prevention and Treatment of AIDS and Sexually Transmitted Diseases, Center for Disease Control and Prevention of Xihu District, Hangzhou, Zhejiang, China; 19 International medical center, First Affiliated Hospital, Zhejiang University School of Medicine, Hangzhou, Zhejiang, China; 20 Department of Infectious Disease, First Affiliated Hospital, Zhejiang University School of Medicine, Hangzhou, Zhejiang, China; 21 Department of Disease Control, Center for Disease Control of Jianggan District, Hangzhou, Zhejiang, China; 22 Department of Laboratory, Center for Disease Control of Jianggan District, Hangzhou, Zhejiang, China; 23 Beijing ViewSolid Biotechnology, Beijing, China; 24 Shanghai OE Biotech. Co., Ltd, Beijing, China; 25 Peking-Tsinghua Center for Life Sciences, PKU-IDG/McGovern Institute for Brain Research, Peking University School of Life Sciences, Beijing, China; 26 Netherlands Institute for Neuroscience, Amsterdam, 1105 BA The Netherlands; 27 Institute of Mental Health, National Clinical Research Center for Mental Disorders, Peking University Sixth Hospital, Beijing, China; 28 NHC Key Laboratory of Mental Health, & Research Unit of Diagnosis and Treatment of Mood Cognitive Disorder (2018RU006), Chinese Academy of Medical Sciences, Beijing, China; 29 PKU-IDG/McGovern Institute for Brain Research, Peking University, Beijing, China

**Keywords:** Molecular biology, Developmental biology

## Abstract

Epidemiological studies have demonstrated that the genetic factors partly influence the development of same-sex sexual behavior, but most genetic studies have focused on people of primarily European ancestry, potentially missing important biological insights. Here, we performed a two-stage genome-wide association study (GWAS) with a total sample of 1478 homosexual males and 3313 heterosexual males in Han Chinese populations and identified two genetic loci (rs17320865, Xq27.3, *FMR1NB*, *P*_*meta*_ = 8.36 × 10^−8^, OR = 1.29; rs7259428, 19q12, *ZNF536*, *P*_*meta*_ = 7.58 × 10^−8^, OR = 0.75) showing consistent association with male sexual orientation. A fixed-effect meta-analysis including individuals of Han Chinese (*n* = 4791) and European ancestries (*n* = 408,995) revealed 3 genome-wide significant loci of same-sex sexual behavior (rs9677294, 2p22.1, *SLC8A1*, *P*_*meta*_ = 1.95 × 10^−8^; rs2414487, 15q21.3, *LOC145783*, *P*_*meta*_ = 4.53 × 10^−9^; rs2106525, 7q31.1, *MDFIC*, *P*_*meta*_ = 6.24 × 10^−9^). These findings may provide new insights into the genetic basis of male sexual orientation from a wider population scope. Furthermore, we defined the average ZNF536-immunoreactivity (ZNF536-ir) concentration in the suprachiasmatic nucleus (SCN) as lower in homosexual individuals than in heterosexual individuals (0.011 ± 0.001 vs 0.021 ± 0.004, *P* = 0.013) in a postmortem study. In addition, compared with heterosexuals, the percentage of ZNF536 stained area in the SCN was also smaller in the homosexuals (0.075 ± 0.040 vs 0.137 ± 0.103, *P* = 0.043). More homosexual preference was observed in *FMR1NB*-knockout mice and we also found significant differences in the expression of serotonin, dopamine, and inflammation pathways that were reported to be related to sexual orientation when comparing CRISPR-mediated *FMR1NB* knockout mice to matched wild-type target C57 male mice.

## Introduction

A controversial question in the neurobiology of human behavior relates to the mechanisms underlying sexual orientation. Behavioral traits probably involve complex interactions among genetic, biological, experiential, and sociocultural factors^1^. It has been shown that 2–6% of males are homosexual^2^. Twin studies have shown that male sexual orientation is heritable^3^; and more specifically, the reported estimates of heritability are ~60%^4^. An early exciting observation about the genetics of homosexuality is a study that mapped male sexual orientation to the Xq28 on chromosomal region using microsatellite markers, and especially showing evidence for maternal transmission^5^. And this linkage locus was replicated in one independent sample of males, but not in females, by the same laboratory^1^. However, these findings were not consistently replicated by another study^6^. In addition, studies of two candidate genes, namely, androgen receptor^7^ and aromatase cytochrome P450^8^, have provided negative results. One genome-wide scan of male sexual orientation, with 403 microsatellite markers at 10-cM intervals^9^, showed a combined MLOD score of 3.45 and equal contributions from maternal and paternal allele transmission on 7q36. Two additional regions with a suggestive level of linkage were located near markers D8S505 on 8p12 and D10S217 on 10q26. A GWAS screening for male sexual orientation observed several SNPs with association signals (*P* < 10^−5^) including multiple supporting SNPs on chromosomes 13 (minimum *P* = 7.5 × 10^−7^) and 14 (*P* = 4.7 × 10^−7^)^10^. Most recently, researchers using large-scale datasets on 477,522 individuals also revealed five loci that are significantly associated with same-sex behavior and concluded that all tested genetic variants accounted for 8% to 25% of the variation in same-sex sexual behavior^11^.

Here, we report a two-stage GWAS of male sexual orientation, with a total of 1478 homosexual men and 3313 heterosexual men in the Han Chinese population (first-stage: 521 homosexual and 1270 heterosexual men; and second-stage: 957 homosexual and 2043 heterosexual men). Then, we performed a genome-wide meta-analysis of the Chinese and European populations (*n* = 413,786). We also used postmortem material from the Netherlands Brain Bank (NBB) to further verify our discovery of the *ZNF536* gene. To determine whether *ZNF536* is expressed at the protein level, the human hypothalamus, which is important for sexual behavior, was stained immunocytochemically. The level of expression was quantified in the SCN, a brain area that is twice as large in homosexual men as in heterosexual men^12^. Since the SCN is thus far the only hypothalamic nucleus related in volume and vasopressin cell number to sexual orientation and not to gender^13^, this nucleus was chosen for the expression study. Finally, we explored the potential biological mechanisms of the associated gene fragile X mental retardation 1 neighbor (*FMR1NB*) gene using CRISPR-mediated knockout mice and bioinformatics analysis. The whole workflow chart of our study is shown in Supplementary Fig. S1.

## Results

### GWAS of sexual orientation in Han Chinese population

**Fig. 1:**
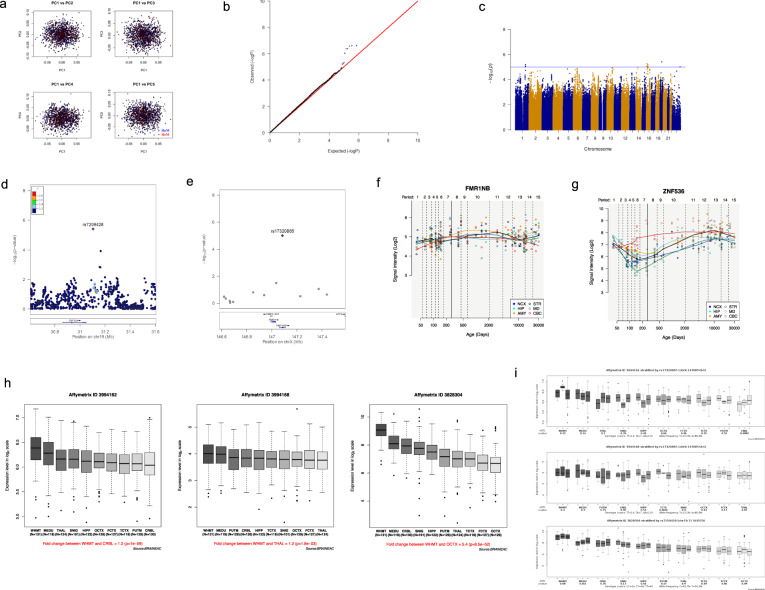
Genome-wide association analysis of the Han Chinese population. **a** Principal component analysis of homosexual men versus heterosexual men. **b** Quantile-quantile plots of the association results of 521 homosexual men and 1270 heterosexual controls (inflation factor *λ*_GC_ = 1.02) from the discovery phase. Results. Overall association results from the discovery phase. **c–e** Manhattan plots of *P* values derived from Cochran-Armitage trend tests in the discovery phase, with 521 homosexual men and 1270 heterosexual controls (**c**); regional plots of the two loci (rs17320865 and rs7259428) associated with male sexual orientation (**d**, **e**). **f** The expression profile of *FMR1NB* using the HBT database. **g** The expression profile of ZNF536 using the HBT database. **h** Differences in the expression levels of *FMR1NB* and *ZNF536* transcripts across various 10 brain regions by using the BRAINEAC database (ID 3994162; ID 3994168; ID 3828304). **i** Difference of expression level of FMR1NB and ZNF536 transcripts across 10 various brain regions by using the BRAINEAC database (ID 3994162; ID 3994168; ID 3828304).

To identify genetic loci associated with sexual orientation, we conducted a genome-wide association analysis in 522 homosexual men and 1294 heterosexual men using Illumina Human Omni-Zhonghua-8 Bead Chip arrays. To perform quality control, single nucleotide polymorphisms (SNPs) were excluded if they had a call rate <98%, minor allele frequency (MAF) <0.01, or significant deviation from Hardy–Weinberg equilibrium (*P* < 1.0 × 10^−4^) in the heterosexual men, and samples were removed because of discordant sex, call rate <95% or duplication, and first- or second-degree relatives. After quality control, 724,550 SNPs in 521 homosexual men and 1270 heterosexual men were retained for the primary GWAS. Genotypes were imputed against the 1000 Genomes Project reference panel^14^ by using IMPUTE2^15^. Then, we performed a GWAS analysis of male sexual orientation using top age and five principal components as covariates. A genomic control inflation factor (*λ*_GC_ = 1.02) also confirmed the minimal evidence for population stratification (Fig. 1a, b). However, no SNPs reached genome-wide significance (*P* < 5 × 10^−8^; Fig. 1c) in primary GWAS. In total, 14 SNPs achieved the suggestive significance level (*P* < 1 × 10^−5^). After linkage disequilibrium (LD) clumping (*r*^2^ > 0.1 and 500-kb windows), 4 LD-independent suggestive associated SNPs were retained for the second-stage analysis. To replicate the results of the primary GWAS, we examined the 4 suggestive SNPs for association with male sexual orientation in an independent cohort of 957 homosexual men and 2043 heterosexual men (Fig. 1c, e and Table 1). All four SNPs showed consistent association in the independent validation samples, and two SNPs reached a genome-wide significance level (*P* < 5 × 10^−8^) after meta-analysis, including SNP rs7259428 on chromosome 19q12 (located in *ZNF536*, *P* = 7.58 × 10^−8^, OR = 0.75; Fig. 1e and Table 1) and rs17320865 on chromosome Xq27.3 (located in *FMR1NB*, *P* = 8.36 × 10^−8^, OR = 1.29; Fig. 1d and Table 1). For the two genome-wide significant loci, we used the Bayesian method to identify the credible sets of variants at each locus most likely to include a variant with causal effect^16^. Two loci were fine-mapped, including 19q12 (index variant rs7259428) and Xq27.3 (index variant rs17320865) (Table 1).

**Table 1:** Evidence of an association of the four SNPs in a two-stage GWAS in Han Chinese population.

SNP	CHR	BP	A1/A2	Gene	Discovery stage (521 homosexual and 1270 heterosexual men)			Replication stage (957 homosexual and 2043 heterosexual men)			Meta-analysis (1478 homosexual and 3313 heterosexual men)		
					OR	SE	*P*	OR	SE	*P*	OR	*P*	P_Het
rs17320865	X	147085464	A/T	*FMR1NB*	1.39	0.07	9.77 × 10^−6^	1.26	0.06	8.81 × 10^−4^	1.31	7.61 × 10^−9^	0.30
rs7259428	19	31104579	G/A	*ZNF356*	0.67	0.09	3.87 × 10^−6^	0.64	0.07	1.13 × 10^−6^	0.65	8.20 × 10^−15^	0.69
rs12039940	1	181952711	C/T	*ZNF648*	1.57	0.10	9.22 × 10^−6^	1.01	0.04	8.72 × 10^−1^	1.07	6.28 × 10^−2^	1.0 × 10^−4^
rs7500300	16	5932886	C/T	*RBFOX1*	1.45	0.08	5.28 × 10^−6^	1.11	0.05	6.84× 10^−2^	1.19	3.08 × 10^−5^	5.2 × 10^−3^

*CHR* chromosome, *OR* odds ratio, ORs were calculated according to the minor allele, *A1/A2* minor/major allele; *P_Het* *p* values of heterogeneity test.

**Table 2:** Clinicalpathological details of postmortem patients.

NBB	Autopsy	Sex	Age	BW (g)	PMD (h)	FT (d)	Death time	Death month	Time in papaffin (y)	AIDS	Diagnosis
Heterosexual men (*n* = 13)											
94–040	94/019	m	20	1490	41.00	82	8:30	1	26	N	Heart failure; Fibronous hemorrhagic pneumonia; B-cell lymphoma
89–024	89/059.2	m	21	1430	49.00	25	16:00	2	31	Y	AIDS; mycobacterial infections; pneumonia; cerebrovascular accident
82–020	82/181	m	27	1560	41.00	40	0:00	7	38	N	Drug addiction; sepsis; cerebral edema
86–042	86/414.1	m	28	1450	17.00	46	0:00	11	34	N	Guilian–-Barré syndrome; bronchopneumonia
89–042	86/116.2	m	30	1340	29.75	25	4:15	4	31	Y	AIDS; disseminated non–Hodgkin lymphoma infections; drug use
86–048	86/436.5	m	30	1430	8.83	37	1:10	12	34	Y	AIDS; pneumocystis carinii pneumonia; lung tuberculosis; toxoplasmosis
97–075	97/096.	m	33	1410	18.75	32	14:15	5	23	N	Brain damage following motor accident
98–031	97/406	m	33	1588	18.42	72	18:35	12	23	N	Status post traffic accident
98–144	98/156.	m	36	1710	17.67	33	19:20	9	22	Y	HIV-encephalopathy
87–069	87/345.1	m	41	1440	99.98	31	0:00	10	33	N	Cerebral contusion; lung emboli
88–011	88/039.1	m	41	1500	20.50	33	13:30	1	32	N	Fortral and benzodiazepine intoxication; heart failure
93–072	93/026	m	50	1573	9.00	52	8:00	1	27	N	Hypovolemic shock
95–102	95/310	m	53	1383	10.00	33	7:30	11	25	N	type A dissection of the ascending aorta
homosexual men (*n* = 13)											
89–031	89/079.3	m	25	1530	23.00	28	9:30	3	31	Y	AIDS, pneumonia
88–009	88/030.5	m	30	1480	4.93	27	10:04	1	32	Y	AIDS; cytomegalic infections; active euthanasia
86–046	86/426.1	m	32	1440	48.83	11	9:10	12	34	Y	AIDS; pneumocystic carinii pneumonia
89–109	89/7898.	m	32	1340	9.50	134	2:00	2	31	Y	AIDS
86–038	86/398.1	m	37	1210	\	5	0:00	1	34	Y	AIDS
89–084	89/262.5	m	39	1670	41.00	28	0:00	8	31	Y	AIDS; Kaposi’s sarcoma
87–080	87/396.5	m	39	1320	89.00	28	0:00	11	33	Y	AIDS; progressive multifocal leukoencephalopathy
87–084	87/466.2	m	40	1520	17.00	27	0:00	12	33	Y	AIDS
87–030	87/159.5	m	41	1520	41.00	40	0:00	5	33	Y	AIDS; respiratoire insufficientie
88–087	88/285.0	m	41	1240	8.17	34	8:50	8	32	Y	AIDS; bronchopneumonia; cytomegalic infections; toxoplasmosis
86–043	86/415.6	m	42	1340	\	24	0:00	1	34	Y	AIDS; Karposi’s sarcoma; generalized mycobacterium avium infections
88–121	88/384.5	m	42	1340	18.75	30	13:45	11	32	Y	AIDS; cytomegalic meningoencephalitis
86–023	86/262.5	m	43	1260	2.00	100	0:00	8	34	Y	AIDS; disseminated Karposi’s sarcoma; pneumonia
p-value			0.227	0.105	0.621	0.098	0.253	0.154	0.073		

*NBB* patients number of the Netherlands Brain Bank, *m* male, *BW* brain weight, *PMD* postmortem delay, *FT* fixation time, *g* grams, *h* hours, *d* days, *y* years, *Y* yes, *N* no, *AIDS* Acquired Immune Deficiency Syndrome, *AZT* azidothymidine, *DHPG* dehydroxy-phenylglycol.

### Genome-wide meta-analysis of the Chinese and European populations

To further increase the statistical power, we performed a meta-analysis of our primary GWAS and European GWAS^17^. The European GWAS of sexual orientation consisted of two datasets: (i) GWAS conducted in males only and (ii) GWAS conducted in males and females combined. In the meta-analysis of the GWAS conducted in males, we detected 50 SNPs surpassing the genome-wide significance threshold for association with sexual orientation (Supplementary Table S1). After clumping the variants by using *r*^2^ > 0.1 and merging the LD-independent variants within 250 kb (Supplementary Table S1), two LD-independent SNPs, namely, rs9677294 (2p22.1, *SLC8A1*, *P* = 1.95 × 10^−8^) and rs2414487 (LOC145783, *P* = 4.53 × 10^−9^) were identified. Then, we combined our primary GWAS with the full European GWAS. In total, 16 SNPs reached a genome-wide significance level in the joint analysis (Supplementary Table S2). After clumping, one LD-independent SNP, rs2106525 (*MDFIC*, *P* = 6.24 × 10^−9^) was retained (Supplementary Table S2).

### Polygenic risk-score profiling

**Fig. 2:**
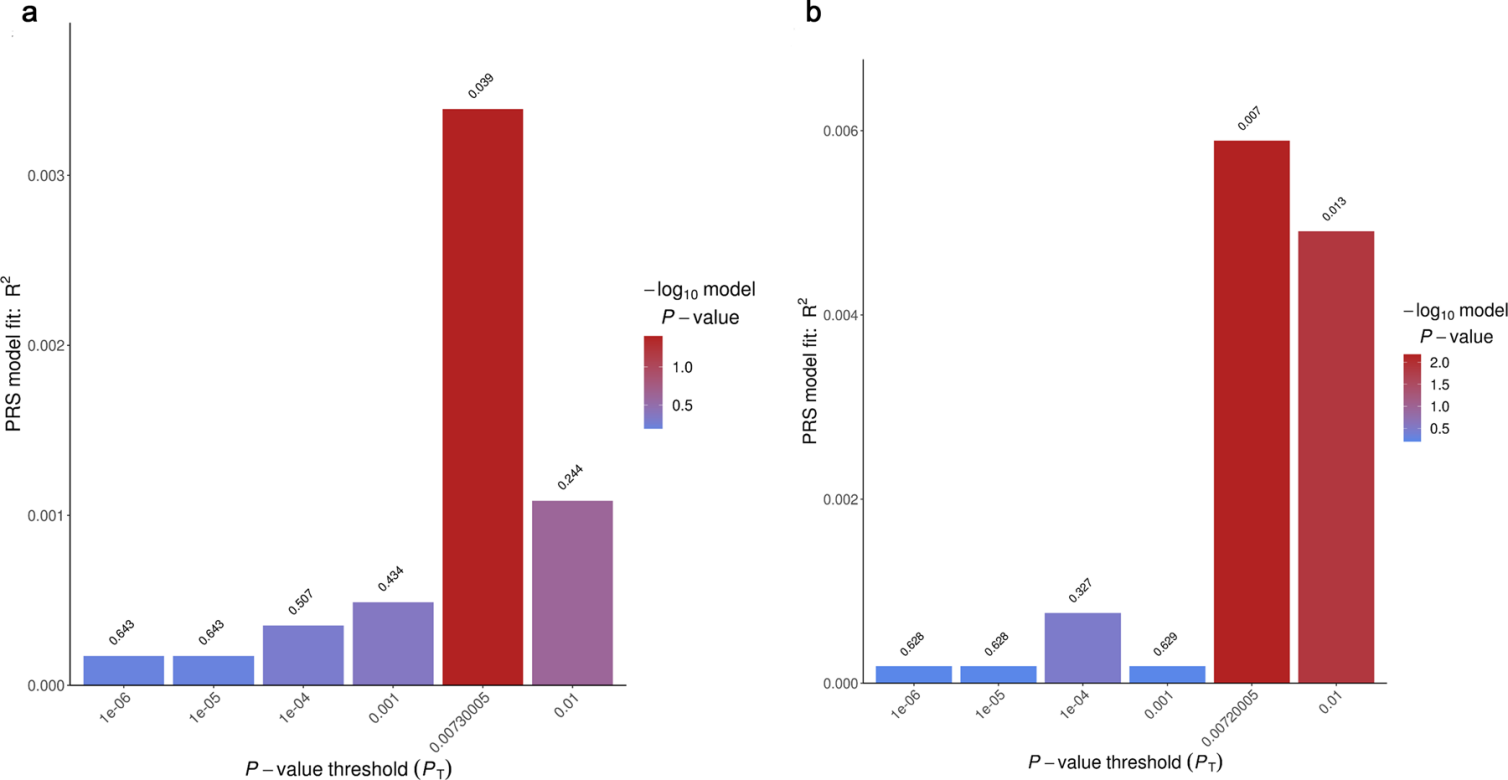
Ploygenic risk score profiling using the European results as the discovery set and the Chinese data as the testing set. The *x* axis shows six *P* value thresholds (*P* = 1e-6, 1e-5, 1e-4, 1e-3, and 1e-2) and the best-threshold (*P* = 7e-3), which showed the maximal Nagelkerke *R2*. The *y* axis shows the Nagelkerke *R2*, i.e., the proportion of variance in case-control status explained by the risk score profile. The number above each bar is the *P* value for the capacity of the risk score profile to predict case-control status for that PT. **a** The GWAS results of males as the discovery set. **b** The GWAS conducted in males and females combined as the discovery set.

To examine the genetic overlap of sexual orientation between the European and Chinese populations, we conducted a polygenic scoring analysis using PRSice2^18^. We used the Chinese sample as the target sample and the European GWAS as the training dataset. We observed improved predictive performance when the *P*-value threshold was gradually increased (Fig. 2). The genetic risk scores based on European GWAS showed a significant capacity to predict case–control status in the Han Chinese samples (Fig. 2). The risk-profile SNPs from the European GWAS data explained ~1.11%–2.34% of the variance in the case–control status of the Chinese sample on the liability scale (Fig. 2).

### Potential biological mechanisms of the associated loci

To test the biological plausibility of two identified genes, *FMR1NB* and *ZNF536*, for male sexual orientation, we investigated expression enrichment profiling of the polymorphic genes in human brain tissues using public databases, including Human Brain Transcriptome (HBT)^19^ and BRAINEAC^20^. Using the HBT database, we observed that *FMR1NB* and *ZNF536* mRNAs were preferentially expressed in human brain tissues such as the whole brain, frontal cortex, and subthalamic nucleus, all of which showed higher enrichment scores. The expression level of *FMR1NB* was relatively higher in the middle stage of life (Fig. 1f). Temporal expression analyses showed that the level of *ZNF536* expression was relatively high in the early fetal and late stages of life (Fig. 1g). The results of gene expression analysis further supported the putative role of *FMR1NB* and *ZNF536* in regulating physical function. Using the BRAINEAC database, we also explored the difference in *FMR1NB* and *ZNF536* mRNA expression across 10 various brain regions. *FMR1NB* [transcript ID 3994162] was differentially expressed between the intralobular white matter (WHMT) and cerebellar cortex (CRBL) (fold change = 1.2, *P* = 1.0 × 10^−9^), transcript ID 3994168 was differentially expressed between the WHMT and thalamus (fold change = 1.2, *P* = 1.8 × 10^−3^) (Fig. 1h); and the *ZNF536* [transcript ID 3828304] was also highly differentially expressed between the WHMT and occipital cortex (OCTX), with a fold change of 5.4 (*P* = 8.5 × 10^−52^) (Fig. 1h).

To understand the molecular function of these genetic associations, we conducted an analysis of expression quantitative trait loci (eQTLs) using the gene expression data of postmortem human brains (*n* = 131) from the BRAINEAC database^20^. Moreover, the intronic SNP rs17320865 at *FMR1NB* showed significant *cis*-eQTL effects on its transcript ID 3994162 in CRBL (*P* = 0.0082) and on another transcript ID 3994168 in temporal cortex (TCTX) (*P* = 0.0041) (Fig. 1i). The most significant SNP at *ZNF536* (rs7259428) was found to be associated with the expression level of its transcript ID 3828304 in the mesencephalon (for exon-specific probes of mRNA expression in the mesencephalon (*P* = 0.011) (Fig. 1i).

### ZNF536 staining in the hypothalamus

ZNF536 staining was observed in the hypothalamus neurons, stronger in the cytoplasm than in the nucleus, in astrocytes, endothelial cells, and the nuclei of ependymal. Predominant cytoplasmic ZNF536-ir was observed in the neurons of the paraventricular nucleus, the super optic nucleus, the nucleus basalis of Meynert, and the zone incerta.

**Fig. 3:**
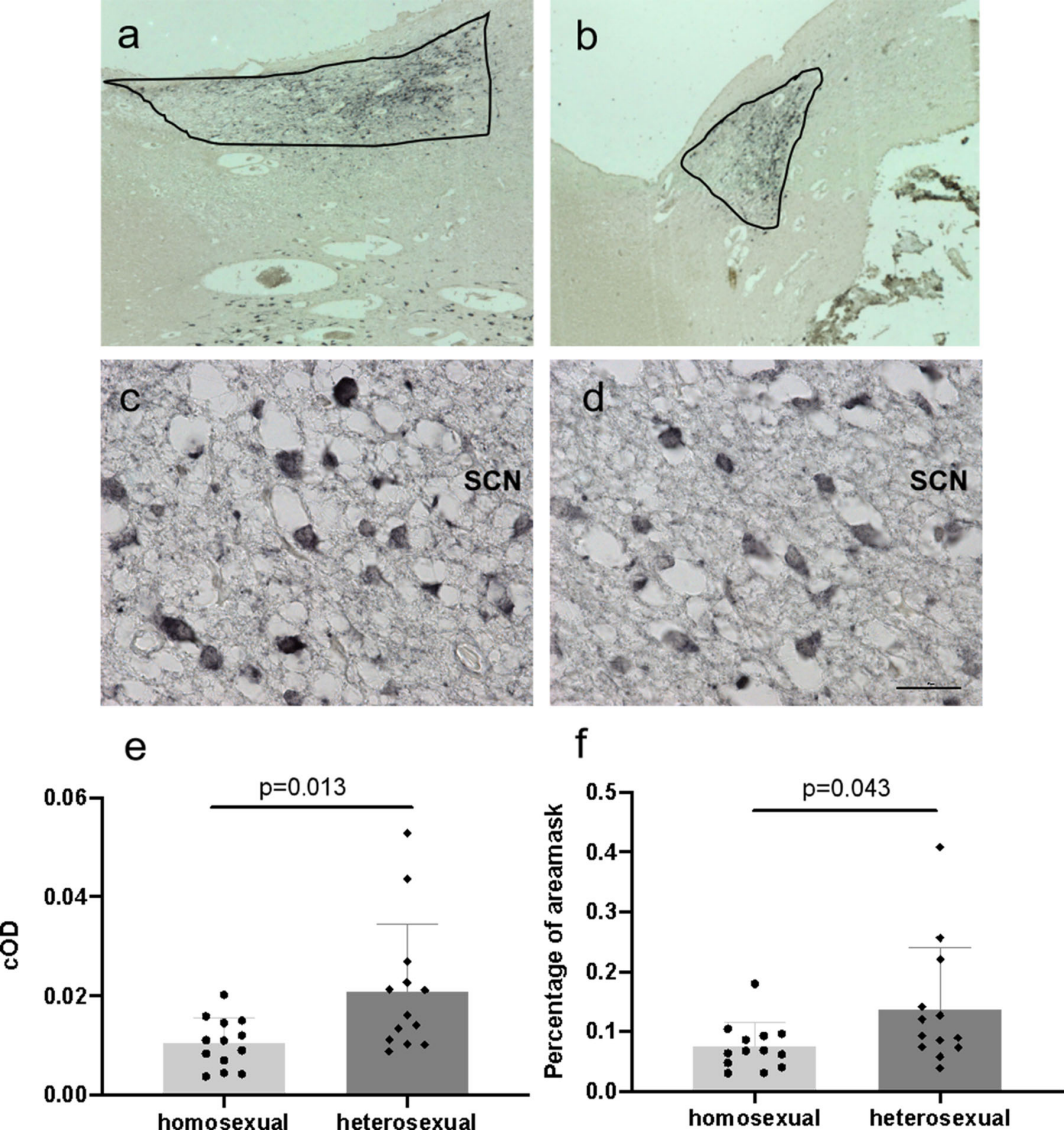
Postmortem study of *ZNF536*. **a**, **b** AVP-immunoreactivity (AVP-ir) is identified in the SCN of a homosexual man (**a**) and a heterosexual man (**b**). **c**, **d** ZNF536-immunoreactivity (ZNF536-ir) of a heterosexual man (**c**) and a homosexual man (**d**) in the area of the SCN. Data are presented as the means ± SEM. **e**, **f** The cOD of ZNF536 was significantly lower level in homosexual subjects (*n* = 13) than in heterosexual subjects (*n* = 13) (**e**), and the percentage of stained area was relatively smaller in homosexual subjects (**f**).

The anti-vasopressin (AVP)-stained SCN area of homosexual subjects was larger than that of heterosexual males (0.81 ± 0.33 mm^2^ vs 0.54 ± 0.24 mm^2^; *P* = 0.026, Fig. 3a, b), in accordance with our earlier work^12^. ZNF536 was present in the cytoplasm and nucleus of SCN neurons and glial cells, both in homosexual and heterosexual subjects (Fig. 3c, d). The corrected optical density (cOD) of ZNF536 was significantly lower level than in homosexual subjects compared with heterosexual subjects (homosexuals: 0.011 ± 0.001; heterosexuals: 0.021 ± 0.004, *P* = 0.013, Fig. 3e). The percentage of stained area was relatively smaller in homosexual subjects (homosexuals: 0.075 ± 0.040; heterosexuals: 0.137 ± 0.103, *P* = 0.043, Fig. 3f).

### Animal sexual behavior associated with the FMR1NB gene

**Fig. 4:**
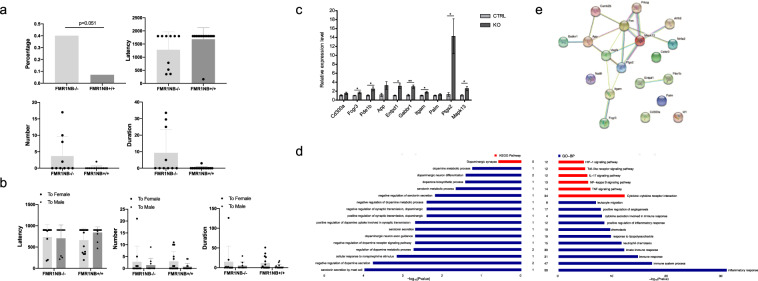
Animal sexual behavior and transcriptomics associated with the *FMR1NB* gene. **a** The mounting behavior of *FMR1NB*^−/−^ (*n* = 10) and *FMR1NB*^*+/+*^ (*n* = 14) male mice in resident-intruder tests: The percentage of males who mounted target males was higher in *FMR1NB*^−/−^ males than *FMR1NB*^*+/+*^ males. The difference was very nearly significant (*P* = 0.051). *FMR1NB*^−/−^ male mice mounted with a tendency for a higher frequency (*P* = 0.092) and longer duration (*P* = 0.071); Sexual preference of *FMR1NB*^*+/+*^ and *FMR1NB*^−/−^ male mice in the mating choice assay. **b** The mounting latency, number and duration toward females in *FMR1NB*^*+/+*^ and *FMR1NB*^−/−^ male mice are shown. **c** Relative expression level of DEGs. **d** Altered GO and KEGG pathways of serotonin, dopamine (left) and representative inflammatory (right) DEGs. **e** Protein-Protein Interaction (PPI) Network of DEGs.

To further identify the biological function of the FMR1NB gene, we established CRISPR-mediated FMR1NB knockout (*FMR1NB*^*−/−*^) mice. No significant morphological changes (including weight and litter size) were observed between *FMR1NB*^−/−^ and *FMR1NB*^*+/+*^ male mice. We examined how male mice responded in their home cage when a wild-type target C57 (*FMR1NB*^+/+^) male mice were introduced. Compared to the *FMR1NB*^*+/+*^, *FMR1NB*^*−/−*^ male mice showed a tendency to mount more male intruders. The percentage of males who mounted target males was higher in *FMR1NB*^−/−^ males than *FMR1NB*^*+/+*^ males. The difference was nearly significant (*P* = 0.051). *FMR1NB*^−/−^ male mice mounted with trends of a higher frequency (*P* = 0.092) and a longer duration (*P* = 0.071). These results suggested that the absence of FMR1NB protein in the brain might increase male-male mounting. Another important result showed a change in the sexual preference of *FMR1NB*^−/−^ male mice. In the mating choice assay, *FMR1NB*^*+/+*^ males significantly preferred to mount females. However, the mounting latency, number, and duration toward females of *FMR1NB*^−/−^ male mice were not significantly different from those toward males. These results are exhibited in Fig. 4a.

Then, we investigated the RNA-Seq gene network modules between two CRISPR-mediated *FMR1NB* knockout mice and two matched wild-type target C57 male mice. According to the chi-square test or *t* test, there was no difference in age or sex between the CRISPR-mediated *FMR1NB* knockout mice and matched wild-type target C57 male mice (*P* > 0.05). Overall, 795 out of 21,879 identified genes showed significant statistical differences. Among these differentially expressed genes (DEGs), four genes (*Arrb2*, *Cd300a*, *Fcgr3*, and *Pde1b*) were associated with serotonin metabolic processes, which were all upregulated genes. Additionally, 18 DEGs (*Celsr3, Dlg4, Entpd1, Gabbr1, Itgam, Nr4a2, Palm, Ptgs2, Vangl2, Camk2b, Fos, Gnb2, Kif5a, Mapk13, Ppp2r3d*, and *Prkcg*) were associated with dopamine metabolic processes. Except for *Vangl2* and *Camk2b*, which were downregulated, the rest of the DEGs were all up-regulated genes. Details are shown in Supplementary Table S3. Further verification of the expression quantity of some DEGs is shown in the supplementary material (Fig. 4c and Supplementary Table S3). Intriguingly, we also found a significant difference in the expression of a large number of inflammation-related genes and pathways between the CRISPR-mediated *FMR1NB* knockout mice and matched wild-type target C57 male mice. We chose the most representative gene ontology (GO) terms and KEGG (http://www.genome.jp/kegg/) pathways, as shown in Fig. 4d. For serotonin and dopamine metabolic processes, DEGs between the CRISPR-mediated FMR1NB knockout mice and matched wild-type target C57 male mice were significantly enriched in 21 GO terms (19 GO terms for dopamine, 4 GO terms for serotonin and 2 terms are shared by both) and one KEGG pathway (*P* < 0.05 Fig. 4d). Moreover, we used the STRING online tool to construct the Protein–Protein Interaction Network of DEGs (Fig. 4e).

## Discussion

The top significantly-associated SNP, rs17320865, is found in an intron of the *FMR1NB* gene. *FMR1NB* has been reported to play a role during brain development, which makes it a potential candidate for the development of dyslexia^21,22^. There was no previous evidence of an association between *FMR1NB* and sexual preference. In the present study, adult *FMR1NB* knockout males showed a near-significant trend of male-male mounting. The results suggested that the *FMR1NB* gene was potentially associated within male sexual orientation. However, the error bars of the *FMR1NB*^−/−^ group were rather large, which may result from individual variation and a relatively small sample size.

Moreover, the peripheral blood transcriptomics results indicated significant differences in the expression of serotonin, dopamine, and inflammation, which were reported to might be related to sexual preferences, between the CRISPR-mediated *FMR1NB* knockout mice and matched wild-type target C57 male mice. A previous study^23^ reported that mice lacking central serotonergic neurons lost sexual preference. Another finding of transcriptomics is the alteration of the dopamine metabolic process. Moreover, an appreciable quantity of inflammatory pathways was changed in our transcriptome results.

The other top significantly associated SNP, rs7259428, is found in an intron of the *ZNF536* gene. As a member of zinc finger protein, which defines a large family of transcription factors, *ZNF536*, is found to be most abundant in the brain and is especially expressed in the dorsal root ganglia, cerebral cortex, hippocampus, and hypothalamic area^24^. It is of interest to note that the average concentration of ZNF536-ir was significantly lower in the SCN of homosexual individuals than in that of heterosexual individuals, although the total amount did not differ between the two groups.

We also compared our Chinese GWAS with the European GWAS using polygenic risk score profiling. Our results reveal that there are shared genetic components of sexual orientation susceptibility across the world populations. There is also evidence for many population differences in risk alleles. For European populations, previous genetic linkage studies reported genome-wide significant linkage to chromosomes 8^25^, 13, 14^26^, and chromosome Xq28^5^. However, no previous studies have reported a positive association with chromosomes 19q13 and Xq27.3. These discrepancies might result from different genetic architectures and environmental factors between populations.

The SNPs in previously reported association regions did not show a significant association in the present study (Supplementary Table S4). It is well known that the controversial linkage on Xq28 was hotly argued at the end of the last century^27,28^. However, we did not find a significant association between the SNPs on Xq28 and male homosexuality in the present study. No significant association was found in the SNPs on the reported chromosomes 7q36, 8p12, and 10q26^9^ (Supplementary Table S4).

Due to controversial public opinions on homosexuality research and the complexity in the genetic sense, the progress in the field has been slow and halting. Moreover, genetic association studies regarding male sexual orientation have been sparse, relatively small, mostly based on a primarily European or American ancestry sample or failed to capture the multifaceted richness and complexity of human sexual orientation. Our study, as one of the continued genetic studies on male sexual orientation, would help open a gateway to other studies focusing on genetic and environmental mechanisms of sexual orientation and development. We used Han Chinese populations as a new ancestry sample to perform the GWAS of male sexual orientation and identified two associated loci at Xq27.3 and 19q12. Combining the European and Chinese GWAS results, we also identified 3 genome-wide significant loci at 2p22.1, 7q31.1, and 15q21.3. We also verified the function of the *FMR1NB* gene by the animal behavioral and transcription studies of the gene knockout mice and the *ZNF536* gene by immunohistochemistry of postmortem brain material. Our findings suggest that genetic influences on sexual orientation are highly polygenic and may relate to discovery samples or measures. In summary, our study highlights the importance of genetic factors in male sexual orientation and advances our understanding of the genetic correlates. Moving forward, our efforts and limited resources may be more effectively applied by expanding the sample size and ancestry of the study population.

## Materials and methods

### Participants

In the discovery stage, 522 homosexual men were recruited from the Department of Mental Health at the First Affiliated Hospital of Zhejiang University and 1294 heterosexual men were recruited from the Institute of Mental Health, Peking University. In the replication stage, 957 homosexual men and 2043 heterosexual men were enrolled from the Department of Mental Health at the First Affiliated Hospital of Zhejiang University and from many Centers for Disease Control in Zhejiang Province, China. We assessed the sexual orientation of each participant using the Kinsey scale, which ranges from 0 (denoting exclusively heterosexual orientation) to 6 (for exclusively homosexual orientation), with a score of 3 indicating equally homosexual and heterosexual (bisexual). A score of 4 is defined as predominantly homosexual but more than incidentally heterosexual. A score of 5 is defined as predominantly homosexual and only incidentally heterosexual. The ratings of all controls totaled a score of 0. Those of homosexual men all ranged from 5 to 6 in the discovery and replication stages. In a structured interview, we verified that none of the participants had a history of substance abuse or major illness. No participant reported symptoms of sexual dysfunction, gender identity disorder, or paraphilia, and none had committed sexual offenses. The study was approved by the Ethics Committee of the First Affiliated Hospital, Zhejiang University School of Medicine, and was conducted according to the principles of the Declaration of Helsinki.

For the postmortem study, in total, the hypothalami of 26 male subjects (13 homosexual men and 13 heterosexual men) ranging in age from 20 years to 53 years were obtained by the NBB. Informed consent for a brain autopsy and for the use of the brain material and medical records for research purposes was given by the donor or their next of kin. Clinical-pathological details are given in Table 2. The homosexual and heterosexual subjects were well matched for possible confounding factors, including age, postmortem delay (PMD), fixation time (FT), clock time of death, month of death, and brain weight (BW) (Table 2). Only the storage time in paraffin showed a trend of significance between the two groups (*P* = 0.073). However, since this parameter did not show a significant correlation with the cOD in either homosexual (*P* = 0.179) or heterosexual subjects (*P* = 0.733), it did not affect our results.

### Genotyping and quality control in the GWAS

Genome-wide genotyping of 522 homosexual men and 1294 heterosexual men was performed using the Illumina HumanOmni-Zhonghua-8 Bead Chip according to the manufacturer’s standard instructions. Systemic quality control of 900,015 SNPs in all samples was performed using PLINK 1.90 software^29^. A total of 31 samples were removed for the following reasons: (i) discordant sex (*n* = 1); (ii) call rate <95% (*n* = 0); and (iii) one of a pair of first- or second-degree relatives (PI_HAT > 0.25) or unexpected duplication (the individual with the lower call rate was removed, *n* = 30). SNPs were excluded from further analysis if they (i) had a call rate <95% (*n* = 5061); (ii) had a significant deviation from Hardy-Weinberg equilibrium (*P* < 1 × 10^−4^) in heterosexual men (*n* = 78); had an MAF < 5% in both homosexual men and heterosexual men (*n* = 168,917); and (iii) did not map to autosomal or X chromosomes (*n* = 1409). After quality control, the overall genotyping rate in the remaining individuals and SNPs was 0.9982.

### Principal component analysis

To identify individuals who might have non-Han Chinese ancestry, participants were assessed for population substructure using EIGENSOFT software (https://www.hsph.harvard.edu/alkes-price/software/) with 206 HapMap subjects (60 CEU (Utah residents with ancestry from northern and western Europe), 57 YRI (Yoruba in Ibadan, Nigeria), 45 CHB (Han Chinese in Beijing), and 44 JPT (Japanese in Tokyo)) as the reference panel (https://ftp.ncbi.nlm.nih.gov/hapmap/)^30^. A set of 54,530 common autosomal SNPs with low LD were included in the Principal Component Analysis (PCA). We imposed stringent criteria for removing outliers, and 85 samples were removed from our samples as population outliers. The PCA results confirmed that the remaining samples were of Han Chinese ancestry, were genetically matched between homosexual men and heterosexual controls, and included no genetic outliers (>6 s.d. from the mean for any of the top ten eigenvectors).

### Meta-analysis in Han Chinese and trans-ancestry

Among the SNPs that passed quality control, the most significantly associated markers (*P*_*discovery*_ < 1 × 10^−5^) were selected for the replication study. Xu et al. have reported that there is a north-south subpopulation structure among Han Chinese individuals^31^. To minimize false-positives resulting from subpopulations, we only selected SNPs with similar allele frequencies in northern and southern controls (difference <0.02). The genotypes of the selected SNPs formed a well-defined cluster group in Illumina Genome Studio. The Sequenom Mass Array platform was used for the replication phase. We also performed a meta-analysis of Chinese and European populations. The European GWAS of sexual orientation consisted of two datasets: (i) GWAS only in males (*n* = 188,825) and (ii) GWAS combining males and females (*n* = 408,995). We performed the inverse-variance-weighted meta-analysis with the fixed-effects model.

### Bioinformatics analysis of two loci

To explore the expression patterns of the top significantly associated genes in human tissues, we used HBT (http://hbatlas.org/pages/hbtd)^19^. The HBT database gene search provides dynamic gene expression throughout development and adulthood in the cerebellar cortex (CBC), mediodorsal nucleus of the thalamus (MD), striatum (STR), amygdala (AMY), hippocampus (HIP), and 11 areas of the neocortex (NCX). To detect the functional effects of the risk SNPs in the associated gene, we analyzed their associations with gene expression levels in the BRAINEAC database (http://caprica.genetics.kcl.ac.uk/BRAINEAC/)^20^. The BRAINEAC database consists of 134 neuropathologically normal donors from the MRC Sudden Death Brain Bank in Edinburgh and Sun Health Research Institute. Gene expression was profiled on an Affymetrix Exon 1.0 ST array. In the BRAINEAC database, we can examine generated eQTL data for ten human brain regions [CRBL, frontal cortex (FCTX), hippocampus (HIPP), mesencephalon (MEDU), OCTX, putamen (PUTM), substantia nigra (SNIG), TCTX, THAM, and WHMT]. To find weaker but ubiquitous signals in the human brain, the mean expression profile was also calculated across the ten brain regions (referred to as the average-all). Our analysis was based on *cis*-eQTL markers identified in the average-all and ten brain-region-specific analyses downloaded from the authors’ webpage. In the case that duplicate significant eQTL entries (SNPs-genes) were observed, we retained only the more significant entry.

### Postmortem study of ZNF536 in homosexual and heterosexual subjects

#### Immunocytochemistry

After autopsy, the hypothalami were fixed in 4% formaldehyde at room temperature for ~1 month (Table 2), dehydrated, and embedded in paraffin. Serial paraffin sections of 6 μm were cut throughout the hypothalamus using a Leitz microtome. Sections were mounted on SuperFrost/Plus (Menzel, Germany) slides and dried for 48 h on a hot plate at 41 °C followed by 24–36 h in an oven at 37 °C. In the anterior part of the hypothalamus, every 100th section was selected for AVP-immunocytochemical staining to identify the midlevel of the SCN in each subject. Staining details were the same as those described in Wu’s study^32^. The specificity of the antibody anti-AVP antibody (D-7, a gift from Dr. A. Silverman to Dr. F. W. Van Leeuwen, The Netherlands Institute for Brain Research, Amsterdam, The Netherlands) was determined earlier^33^.

After determining the midlevel of the SCN area in each subject, an adjacent section was selected for ZNF536 immunocytochemical staining. Following deparaffinization and rehydration, sections were heated for 10 min at 800 W in a microwave oven in 0.01 M sodium citrate buffer (pH 6.0). To suppress background staining, sections were preincubated in TBS containing 5%-milk powder (w/v), pH 7.6, for 1 h at room temperature (RT), (milk powder from ELK, Campina Melkunie, Eindhoven, The Netherlands). Sections were incubated overnight at 4 °C with a 1: 400 dilution in SUMI of the primary rabbit polyclonal anti-ZNF536 antibody (catalog no. GTX85225, GeneTex, North America). The next day, detection was achieved through subsequent incubations with biotinylated antirabbit antibody (cat. BA-1100, Vector Laboratories, Burlingame, CA) at 1: 400 and ABC at 1: 800 in SUMI for 1 h at RT. Immunosignal was visualized using DAB-nickel.

#### Quantification of immunocytochemical staining

Quantification of ZNF536 was performed using an image analysis procedure described elsewhere extensively^34^. Briefly, Image Proversion 6.3 (Media Cybernetics, Rockville, USA) was used. A black and white camera (SONY XC-77E) was mounted on a microscope (Zeiss Axioskop with Plan-NEOFLUAR Zeiss objectives, Carl Zeiss GmbH, Jena, Germany). The SCN (area total) was outlined by the AVP staining and captured with at a ×20 objective. The outline of the SCN was then transferred to the adjacent ZNF536 stained image. The threshold for the positive signal was set to twice the mean optical density (OD) of the background and a mask of the stained structures was made. The computer determined the OD of the mask and surface area covered by the signal (area mask). Multiplying the OD by the area mask gave the integrated optical density (IOD). Subsequently, the final parameter was the cOD, which was calculated by dividing the IOD by the representative area of the SCN. For each subject, the cOD was used to describe the average concentration of ZNF536-ir in the SCN section.

### Sex preferences of CRISPR-mediated FMR1NB gene knockout mice and bioinformatics analysis

#### Generation of CRISPR-mediated FMR1NB knockout mice

CRISPR-mediated *FMR1NB* knockout mice were produced by Beijing View Solid Biotechnology, China. The linear plasmid pCAG-T7-Cas9 cut by the NotI restriction enzyme was used as the in vitro transcriptional template. After gel purification, Cas9 mRNA was transcribed with the mMESSAGE mMACHINE T7 Ultra Kit (Life Technologies). The *FMR1NB*-g1 and *FMR1NB*-g2 templates were amplified based on the gRNA scaffold using T7 promoter sequence-conjugated primers: T7-*FMR1NB*-g1-FP, T7-*FMR1NB*-g2-FP and gRNA-RP. *FMR1NB*-g1 and *FMR1NB*-g2 were transcribed with a fast-in vitro transcription T7 kit (cat. no. VK010, Beijing View Solid Biotechnology, China) and frozen at 80 °C. Zygotes of C57BL/6 mice (*n* = 90) were injected with Cas9 mRNA and *FMR1NB*-g1 and *FMR1NB*-g2 in M2 media (Millipore) using a FemtoJet micromanipulator (Eppendorf, Germany). After microinjection, zygotes were transferred to pseudopregnant females. All mice were maintained in a specific pathogen-free facility. Tail-derived DNA from 2-week-old newborn mice was genotyped by sequencing the PCR products amplified by the primers: *FMR1NB*-sens and *FMR1NB*-anti (Supplementary Fig. S1 and Table S6).

#### Resident-intruder tests

All test mice were sexually naive. The bedding of the test mice had not been changed for at least 4 days. Intruder mice were 11–13 weeks old, sexually naive, and group-housed C57Bl/6J males. All activities within a test were recorded by an infrared camera (Sony Video Recorder, DCR-HC26C). Mounting latency, mounting frequency and total duration of mounting within 30 min were measured.

#### Mating choice assay

The bedding of test mice was not changed for at least four days. A group-housed sexually naive 11- to 13-week-old C57Bl/6J male and a sexually naive estrous 10-week-old C57B1/6 J female were introduced into the cage of each test male. Each test lasted 15 min after the target mouse was introduced. All activities were recorded by an infrared camera. The latency, frequency, and duration of mounting of male or female targets were analyzed.

#### RNA extraction and library preparation

Whole blood was collected from the mouse orbit, and then leukocytes were isolated for RNA extraction. Total RNA was extracted using the mirVana miRNA Isolation Kit (Ambion) following the manufacturer’s protocol. RNA integrity was evaluated using an Agilent 2100 Bioanalyzer (Agilent Technologies, Santa Clara, CA, USA). The samples with an RNA Integrity Number (RIN) ≥ 7 were subjected to the subsequent analysis. The libraries were constructed using the TruSeq Stranded mRNA LTSample Prep Kit (Illumina, San Diego, CA, USA) according to the manufacturer’s instructions. Then, these libraries were sequenced on the Illumina sequencing platform (HiSeqTM 2500 or Illumina HiSeq XTen), and 125 bp/150 bp paired-end reads were generated.

#### Quality control and mapping

Raw data (raw reads) were processed using Trimmomatic^35^. The reads containing ploy-N and the low-quality reads were removed to obtain the clean reads. Then the clean reads were mapped to the reference genome using hisat2^36^.

#### Gene-level quantification, analysis of DEGs, cluster analysis, GO, and KEGG enrichment

The FPKM^37^ value of each gene was calculated using cufflinks^38^, and the read counts of each gene were obtained by htseq-count^39^. DEGs were identified using the DESeq2^40^ (2012) R package functions estimate Size Factors and nbinom Test. *P* value < 0.05 and fold change >2 or fold change <0.5 were set as the thresholds for significant differential expression. Hierarchical cluster analysis of DEGs was performed to explore gene expression patterns. GO enrichment and KEGG^41^ pathway enrichment analyses of DEGs were respectively performed using R based on the hypergeometric distribution.

#### Transcript-level quantification, analysis of DEGs, cluster analysis, GO and KEGG enrichment

For transcript-level quantification, the FPKM^36^ and read counts values of each transcript (protein_coding) were calculated using bowtie2^42^ and express^43^. DEGs were identified using the DESeq2^40^ (2012) functions estimate Size Factors and nbinom Test. A *P* value < 0.05 and fold change > 2 or fold change < 0.5 were set as the thresholds for significant differential expression. Hierarchical cluster analysis of DEGs was performed to explore transcript expression patterns. GO enrichment and KEG^41^ pathway enrichment analyses of DEGs were respectively performed using R based on the hypergeometric distribution.

#### Gene structure extension and novel transcript identification

The reads were reassembled using StringTie^44^. Then gene structure extension and novel transcript identification were performed by comparing the reference genome and the known annotated genes using cuffcompare software^45^.

#### Alternative splicing analysis and SNP-INDEL calling

Alternative splicing analysis of differentially regulated transcript isoforms or exons was performed using ASprofile^46^. SNPs and INDELs were called using samtools^47^ and bcftools^48^, and the details are shown on the samtools webpage (http://samtools.sourceforge.net/mpileup.shtml). Then snpeff^49^ was used to annotates and predict the effects of variants on genes (such as amino acid changes).

### Further verification of the expression quantity of DEGs

#### RNA extraction

Whole blood was collected from the orbits of five CRISPR-mediated FMR1NB knockout mice and five matched target C57 male mice, and then leukocytes were isolated for RNA extraction. Total RNA was extracted using the mirVana miRNA Isolation Kit (Ambion) according to the manufacturer’s specifications. The yield of RNA was determined using a NanoDrop 2000 spectrophotometer (Thermo Scientific, USA), and the integrity was evaluated using agarose gel electrophoresis stained with ethidium bromide.

#### Real-time quantitative RT-PCR

Quantification was performed with a two-step reaction process: reverse transcription and PCR. Each reverse transcription reaction has two steps. The first step involves mixing 0.5 μg of RNA, 2 μl of 4× gDNA wiper Mix, and the addition of nuclease-free H_2_O to 8 μl. Reactions were performed in a GeneAmp^®^ PCR System 9700 (Applied Biosystems, USA) for 2 min at 42 °C. The second step was to add 2 μl of 5 × HiScript II Q RT SuperMix IIa. Reactions were performed in a GeneAmp^®^ PCR System 9700 (Applied Biosystems, USA) for 10 min at 25 °C; 30 min at 50 °C; 5 min at 85 °C. The 10 μl RT reaction mix was then diluted ×10 in nuclease-free water and held at −20 °C. Real-time PCR was performed using LightCycler^®^ 480 II Real-time PCR Instrument (Roche, Switzerland) with a 10 μl PCR reaction mixture that included 1 μl of cDNA, 5 μl of 2× QuantiFast^®^ SYBR^®^ Green PCR Master Mix (Qiagen, Germany), 0.2 μl of forward primer, 0.2 μl of reverse primer and 3.6 μl of nuclease-free water. Reactions were incubated in a 384-well optical plate (Roche, Switzerland) at 95 °C for 5 min, followed by 40 cycles of 95 °C for 10 s and 60 °C for 30 s. Each sample was run in triplicate for analysis. At the end of the PCR cycles, melting curve analysis was performed to validate the specific generation of the expected PCR product. The primer sequences were designed in the laboratory and synthesized by Generay Biotech (Generay, PRC) based on the mRNA sequences obtained from the NCBI database. Sequence details are shown in Supplementary Table S7. The expression levels of mRNAs were normalized to genes (*Arrb2*, *Cd300a*, *Fcgr3*, *Pde1b Celsr3*, *Dlg4*, *Entpd1*, *Gabbr1*, *Itgam*, *Palm*, Ptgs2, *Mapk13*, and *GAPGH*) and were calculated using the 2-ΔΔCt method^50^.

### Statistical analysis

Hardy-Weinberg equilibrium analysis and association analysis of the GWAS phase were conducted using PLINK v1.07^29^. We used Haploview software to generate Manhattan plots and LD structure^51^, and quantile–quantile plots were created using the R program. The regional association plots were drawn using Locus Zoom in a 100-kb window^52^. Allelic association analyses in the replication phase were conducted with the R program. Meta-analyses of GWAS and replication cohorts were performed in R using the Meta package. We used a fixed-effects model if the heterogeneity was not considered significant (*P* value for Cochran’s Q statistic > 0.05); otherwise, we used a random-effects model. The genotype imputation and prephasing steps were performed using IMPUTE2 and SHAPEIT2^53,54^ respectively, and reference data downloaded from the 1000 Genomes Project (1092 individuals, including chromosome X, updated 24-Aug-2012).

In the postmortem study, since the data were not always normally distributed, nonparametric statistics were applied for data analysis. Group comparisons for age, BW, PMD, FT, or time in paraffin were statistically evaluated by the nonparametric Mann-Whitney U test. Correlations were examined with the Spearman test. Differences in the death time and death month were analyzed with the Mardia–Watson–Wheeler test. Differences between the two groups were also analyzed using the nonparametric Mann-Whitney U test with a 5% experimentwise error rate. All tests were two-tailed. A significance of 5% was used in all statistical tests. SPSS 22.0 was applied for the data analysis.

## Supplementary information

Supplementary Information

## Data Availability

All data are available upon request by a qualified researcher.
