# Author Correction: Discovery of new genetic loci for male sexual orientation in Han population

**DOI:** 10.1038/s41421-021-00351-5

**Published:** 2021-11-30

**Authors:** Shao-Hua Hu, Hai-mei Li, Hao Yu, Yan Liu, Chen-Xing Liu, Xian-bo Zuo, Jing Lu, Jia-Jun Jiang, Cai-Xi Xi, Bo-Chao Huang, Hu-Ji Xu, Jian-Bo Hu, Jian-Bo Lai, Man-Li Huang, Jian-Ning Liu, Dan-Ge Xu, Xi-Chao Guo, Wei Wu, Xin Wu, Lei Jiang, Meng Li, Guang-Ping Zhang, Jin-Wen Huang, Ning Wei, Wen Lv, Jin-Feng Duan, Hong-Li Qi, Chan-Chan Hu, Jing-Kai Chen, Wei-Hua Zhou, Wei-Juan Xu, Chen-Feng Liu, Hai-Yong Liang, Jing Du, Shu-Fa Zheng, Qiao-Ling Lu, Lin Zheng, Xiao-Wei Hu, Feng-Xiang Chen, Peng Chen, Biao Zhu, Li-Jun Xu, Zhi-Min Ni, Ye-Zhen Fang, Zuo-Kai Yang, Xin-Ren Shan, En-de Zheng, Fan Zhang, Qing-qing Zhou, Yi Rao, Dick Swaab, Wei-Hua Yue, Yi Xu

**Affiliations:** 1 Department of Psychiatry, First Affiliated Hospital, Zhejiang University School of Medicine, Hangzhou, Zhejiang China; 2 The Key Laboratory of Mental Disorder Management in Zhejiang Province, Hangzhou, Zhejiang China; 3 National Human Brain Bank for Health and Disease, Hangzhou, Zhejiang China; 4 Brain Research Institute, Zhejiang University, Hangzhou, Zhejiang China; 5 Department of Psychiatry, Jining Medical University, 133 Hehua Rd, Jining, Shandong China; 6 Department of Neurobiology, School of Basic Medical Sciences, Capital Medical University, Beijing, China; 7 Department of Human Genetics, University of Pittsburgh, Pittsburgh, PA USA; 8 Institute of Dermatology and Department of Dermatology at First Hospital, Anhui Medical University, Hefei, Anhui China; 9 Department of General Office, Center for Disease Control of Jianggan District, Hangzhou, Zhejiang China; 10 Department of Clinical Laboratory, First Affiliated Hospital, Zhejiang University School of Medicine, Hangzhou, Zhejiang China; 11 State Key Laboratory for Diagnosis and Treatment of Infectious Disease, First Affiliated Hospital, Zhejiang University School of Medicine, Hangzhou, Zhejiang China; 12 Department of Rheumatology and Immunology, Shanghai Changzheng Hospital, The Second Military Medial University, Shanghai, China; 13 Department of Psychiatry, Seventh Hangzhou Hospital, Hangzhou, Zhejiang China; 14 Medicines & Biochemical Products Branch, Zhejiang Medicine & Health Products I/E CO., Ltd, Hangzhou, Zhejiang China; 15 BioMiao Biological Technology (Beijing) Co., Ltd, Beijing, China; 16 Beijing Emei Tongde Technology Development Co., Ltd, Beijing, China; 17 Department of Aids Venereal Disease Control and Prevention, Shaoxing Center for Disease Control and Prevention, Shaoxing, Zhejiang China; 18 Department of Prevention and Treatment of AIDS and Sexually Transmitted Diseases, Center for Disease Control and Prevention of Xihu District, Hangzhou, Zhejiang China; 19 International Medical Center, First Affiliated Hospital, Zhejiang University School of Medicine, Hangzhou, Zhejiang China; 20 Department of Infectious Disease, First Affiliated Hospital, Zhejiang University School of Medicine, Hangzhou, Zhejiang China; 21 Department of Disease Control, Center for Disease Control of Jianggan District, Hangzhou, Zhejiang China; 22 Department of Laboratory, Center for Disease Control of Jianggan District, Hangzhou, Zhejiang China; 23 Beijing ViewSolid Biotechnology, Beijing, China; 24 Shanghai OE Biotech. Co., Ltd, Beijing, China; 25 Peking-Tsinghua Center for Life Sciences, PKU-IDG/McGovern Institute for Brain Research, Peking University School of Life Sciences, Beijing, China; 26 Netherlands Institute for Neuroscience, Amsterdam, 1105 BA The Netherlands; 27 Institute of Mental Health, National Clinical Research Center for Mental Disorders, Peking University Sixth Hospital, Beijing, China; 28 NHC Key Laboratory of Mental Health & Research Unit of Diagnosis and Treatment of Mood Cognitive Disorder (2018RU006), Chinese Academy of Medical Sciences, Beijing, China; 29 PKU-IDG/McGovern Institute for Brain Research, Peking University, Beijing, China

**Keywords:** Molecular biology, Developmental biology

Correction to: *Cell Discovery* (2021) 7:103

10.1038/s41421-021-00341-7, Published online 31 October 2021

In the initial published version of this article^1^, we missed some errors while proofreading the manuscript for the publication. The statistical values in Abstract and Results sections were inconsistent with those in Table 1. Upon careful examination of our original data, the second sentence in Abstract section should be “Here, we performed a two-stage genome-wide association study (GWAS) with a total sample of 1478 homosexual males and 3313 heterosexual males in Han Chinese populations and identified two genetic loci (rs17320865, Xq27.3, *FMR1NB*, *P*_*meta*_ = 7.61 × 10^−9^, OR = 1.31; rs7259428, 19q12, *ZNF536*, *P*_*meta*_ = 8.20 × 10^−15^, OR = 0.65) showing consistent association with male sexual orientation.” On page 2, the second column, the lines 28–35 should be “All four SNPs showed consistent association in the independent validation samples, and two SNPs reached a genome-wide significance level (*P* < 5 × 10^−8^) after meta-analysis, including SNP rs7259428 on chromosome 19q12 (located in *ZNF536*, *P* = 8.20 × 10^−15^, OR = 0.65; Fig. 1e and Table 1) and rs17320865 on chromosome Xq27.3 (located in *FMR1NB*, *P* = 7.61 × 10^−9^, OR = 1.31; Fig. 1d and Table 1).” In Table 1, the gene symbol ‘*ZNF356*’ should be ‘*ZNF536*’*.* The corrected version of the Table 1 is displayed below.

**Table 1:** Evidence of an association of the four SNPs in a two-stage GWAS in Han Chinese population.

					Discovery stage (521 homosexual and 1270 heterosexual men)			Replication stage (957 homosexual and 2043 heterosexual men)			Meta-analysis (1478 homosexual and 3313 heterosexual men)		
SNP	CHR	BP	A1/A2	Gene	OR	SE	*P*	OR	SE	*P*	OR	*P*	*P*_Het
rs17320865	X	147085464	A/T	*FMR1NB*	1.39	0.07	9.77 × 10^−^^6^	1.26	0.06	8.81 × 10^−4^	1.31	7.61 × 10^9^	0.30
rs7259428	19	31104579	G/A	*ZNF536*	0.67	0.09	3.87 × 10^−^^6^	0.64	0.07	1.13 × 10^−6^	0.65	8.20 × 10^15^	0.69
rs12039940	1	181952711	C/T	*ZNF648*	1.57	0.10	9.22 × 10^−6^	1.01	0.04	8.72 × 10^−1^	1.07	6.28 × 10^2^	1.0 × 10^−4^
rs7500300	16	5932886	C/T	*RBFOX1*	1.45	0.08	5.28 × 10^−6^	1.11	0.05	6.84 × 10^−2^	1.19	3.08 × 10^−5^	5.2 × 10^−^^3^

*CHR* chromosome, *OR* odds ratio, ORs were calculated according to the minor allele, A1/A2 minor/major allele, *P_Het* *p* values of heterogeneity test.
