# Supplementary material for: Discovery of new genetic loci for male sexual orientation in Han population
Source: Cell Discov. 2021 Oct 31;7:103. doi: 10.1038/s41421-021-00341-7 (PMC8558329; doi:10.1038/s41421-021-00341-7)
Supplement: Supplementary file 1 — Supplementary Information [file 41421_2021_341_MOESM1_ESM.pdf]

Supplementary Materials for

Discovery of new genetic loci for male sexual orientation in Han population

Shao-Hua Hu[1,2,3,4#], Hai-mei Li[1,2#],Hao Yu[7#], Yan Liu[8#], Chen-Xing Liu[11], Xian-bo Zuo[12], Jing Lu[1,2], Jia-Jun Jiang[1,2],Cai-Xi Xi[1,2],Bo-Chao Huang[1,2],Hu-Ji Xu[15],Jian-Bo Hu[1,2], Jian-Bo Lai[1,2],Man-Li Huang[1,2], Jian-Ning Liu[16], Dan-Ge Xu[17], Xi-Chao Guo[16], Wei Wu[17], Xin Wu[13], Lei Jiang[13], Meng Li[13], Guang-Ping Zhang[16], Jin-Wen Huang[1], Ning Wei[1,2], Wen Lv[18], Jin-Feng Duan[1], Hong-Li Qi[1], Chan-Chan Hu[1], Jing-Kai Chen[1], Wei-Hua Zhou[1,2], Wei-Juan Xu[1,2], Chen-Feng Liu[19], Hai-Yong Liang[20], Jing Du[21], Shu-Fa Zheng[16], Qiao-Ling Lu[22], Lin Zheng[14], Xiao-Wei Hu[16], Feng-Xiang Chen[16], Peng Chen[23], Biao Zhu[24], Li-Jun Xu[24], Zhi-Min Ni[25], Ye-Zhen Fang[26], Zuo-Kai Yang[22], Xin-Ren Shan[22], En-de Zheng[27], Fan Zhang[28], Qing-qing Zhou[28], Yi Rao[8,29], Dick Swaab[6],Wei-Hua Yue[5,9,10*], Yi Xu[1,2,3,4*]

Correspondence to: xuyizju@zju.edu.cn; ryue@bjmu.edu.cn

Fig. S1 workflow chart of the whole study

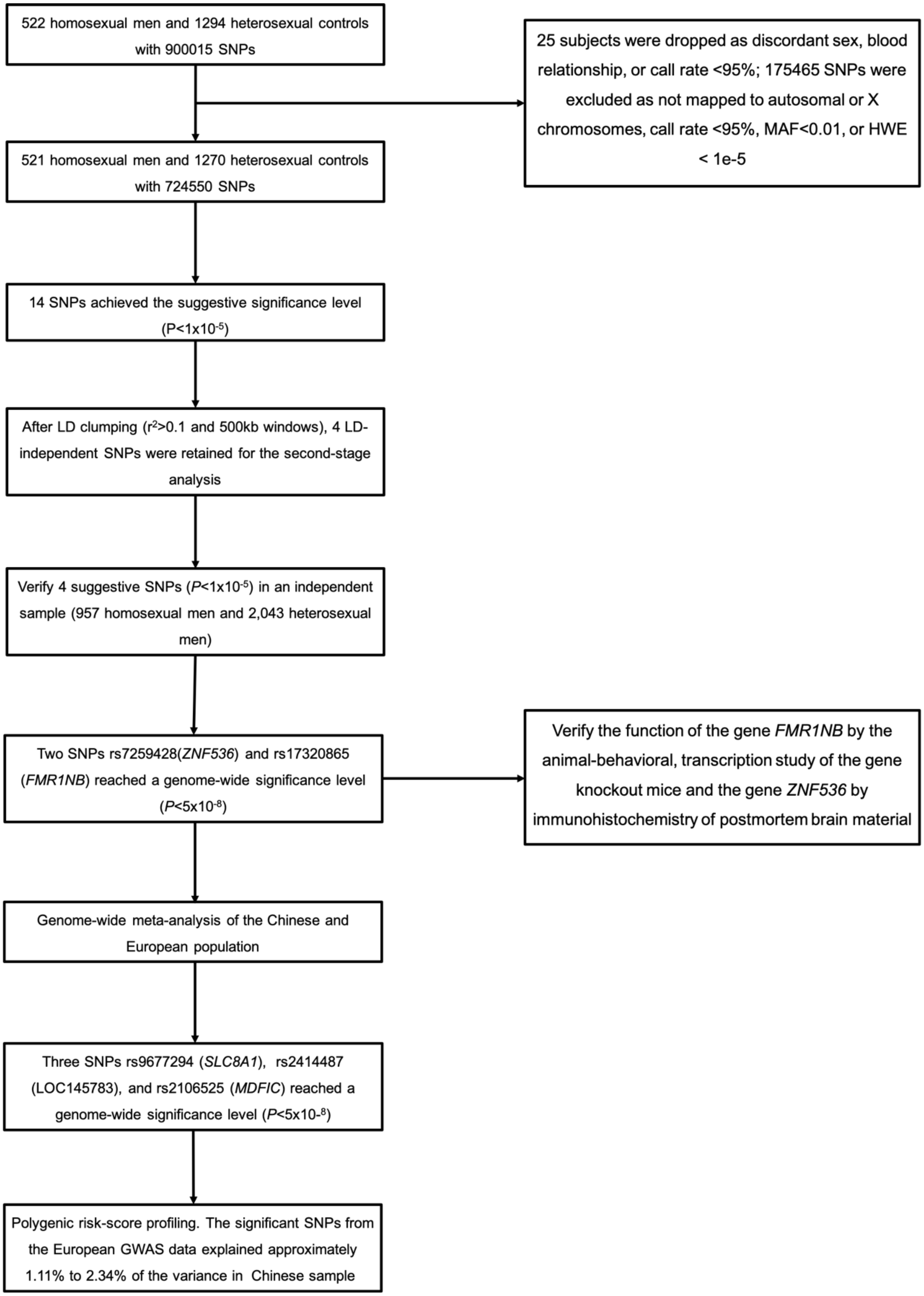

Fig. S2 Generation of CRISPR-mediated FMR1NB knockout mice. (a)Schematic of CRISPR targeting sites within exon1 of the mouse FMR1NB locus. (b) Sanger sequencing was performed on PCR products amplified from tissues of the FMR1NB -/- mouse. (c) sequencing results of the FMR1NB -/- KO mouse, with a terminator "taa" after the CRISPR-mediated, which results in a very short transcript.

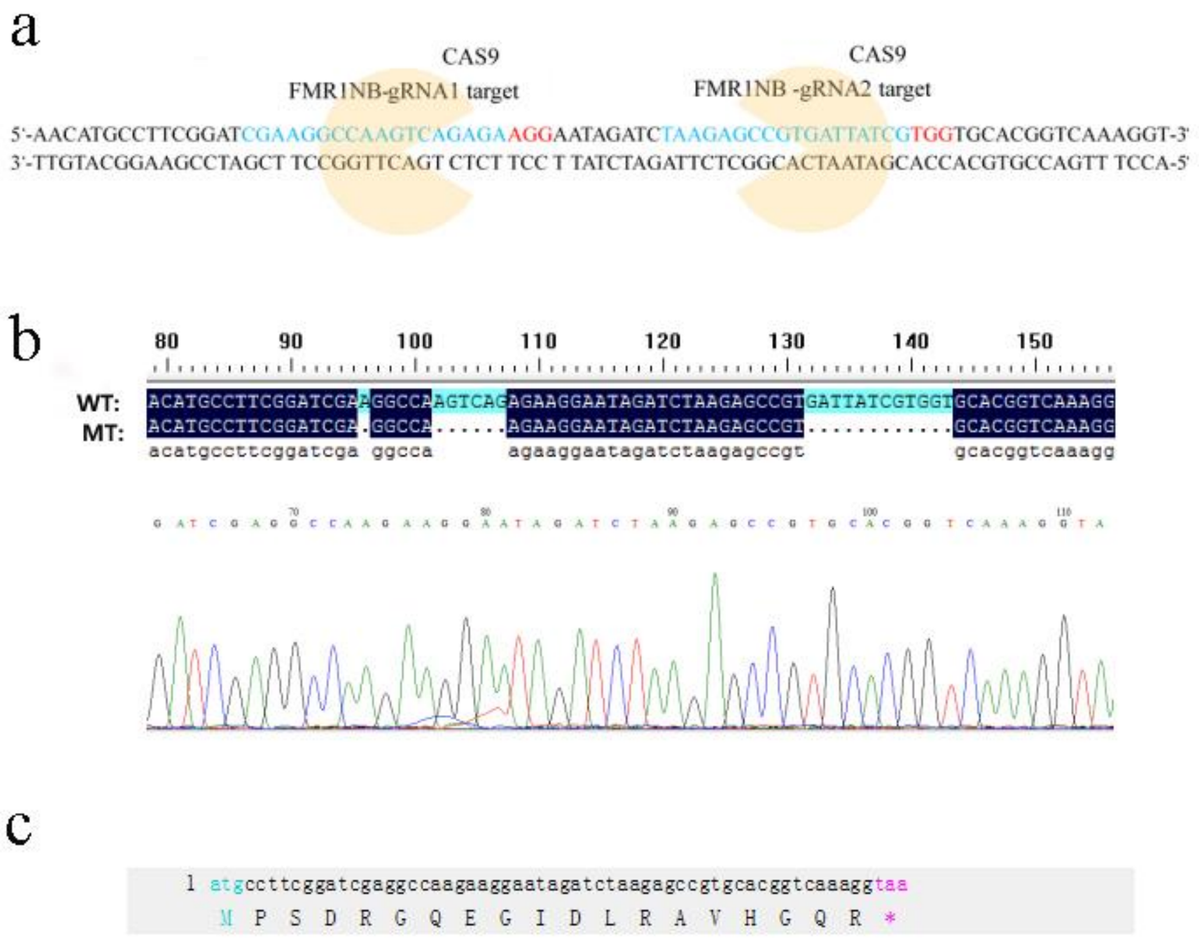

Table S1. Meta-analysis results of male sexual orientation in Chinese and European population.

					Chinese Han population			European population			Meta-analysis			
CHR	SNP	BP	A1	A2	BETA	SE	P	BETA	SE	P	BETA	SE	P	P_heter
2	rs12995661	40849435	C	A	0.075	0.09	3.97E-01	0.004	0.001	3.70E-08	0.004	0.0007	3.62E-08	0.42
2	rs9677294	40849491	T	C	0.060	0.09	4.98E-01	0.004	0.001	2.00E-08	0.004	0.0007	1.95E-08	0.52
2	rs2216039	40863023	G	A	0.085	0.09	3.28E-01	0.004	0.001	5.10E-08	0.004	0.0007	4.90E-08	0.35
2	rs2216038	40863036	T	G	0.089	0.09	3.11E-01	0.004	0.001	4.90E-08	0.004	0.0007	4.67E-08	0.33
2	rs13008134	40863794	G	C	0.099	0.09	2.62E-01	0.004	0.001	4.90E-08	0.004	0.0006	4.70E-08	0.28
15	rs7167847	57167915	T	C	-0.005	0.11	9.67E-01	-0.005	0.001	1.30E-08	-0.005	0.0009	1.27E-08	1.00
15	rs2414487	57172086	A	G	-0.035	0.11	7.41E-01	-0.005	0.001	4.60E-09	-0.005	0.0009	4.53E-09	0.78
15	rs11855646	57178528	A	T	-0.026	0.10	8.00E-01	-0.005	0.001	8.20E-09	-0.005	0.0009	8.10E-09	0.84
15	rs28371400	57188761	A	T	-0.173	0.09	4.39E-02	-0.005	0.001	8.10E-09	-0.005	0.0009	7.20E-09	0.05
15	rs142186994	57197759	T	C	-0.046	0.12	7.07E-01	-0.005	0.001	2.40E-08	-0.005	0.0009	2.41E-08	0.74
15	rs7164655	57238149	C	A	-0.149	0.08	7.85E-02	-0.005	0.001	3.90E-08	-0.005	0.0009	3.51E-08	0.09
15	rs28756149	57248242	T	A	-0.156	0.08	6.34E-02	-0.005	0.001	5.00E-08	-0.005	0.0009	4.54E-08	0.07
15	rs28677775	57261714	G	A	-0.146	0.08	8.37E-02	-0.005	0.001	2.60E-08	-0.005	0.0009	2.33E-08	0.09
15	rs28649108	57264380	A	G	-0.147	0.08	8.13E-02	-0.005	0.001	4.90E-08	-0.005	0.0009	4.40E-08	0.09
15	rs8039899	57288378	C	G	-0.144	0.08	8.86E-02	-0.005	0.001	4.70E-08	-0.005	0.0009	4.25E-08	0.10
15	rs8023816	57288766	G	A	-0.146	0.08	8.45E-02	-0.005	0.001	4.40E-08	-0.005	0.0009	3.95E-08	0.10
15	rs1430822	57294452	G	A	-0.144	0.08	8.86E-02	-0.005	0.001	5.40E-08	-0.005	0.0009	4.90E-08	0.10
15	rs8034066	57295969	G	A	-0.141	0.08	9.41E-02	-0.005	0.001	5.40E-08	-0.005	0.0009	4.86E-08	0.11
15	rs28505122	57325749	A	T	-0.146	0.09	8.67E-02	-0.005	0.001	3.20E-08	-0.005	0.0009	2.93E-08	0.10
15	rs11071303	57333049	C	T	-0.142	0.08	9.27E-02	-0.005	0.001	5.30E-08	-0.005	0.0009	4.85E-08	0.10
15	rs7169053	57338934	A	G	-0.155	0.08	6.74E-02	-0.005	0.001	4.80E-08	-0.005	0.0009	4.31E-08	0.08
15	rs16977222	57344877	C	T	-0.141	0.08	9.53E-02	-0.005	0.001	4.10E-08	-0.005	0.0009	3.70E-08	0.11
15	rs7176873	57349162	A	G	-0.141	0.08	9.34E-02	-0.005	0.001	4.10E-08	-0.005	0.0009	3.70E-08	0.10
15	rs28533433	57354261	C	T	-0.155	0.08	6.53E-02	-0.005	0.001	2.20E-08	-0.005	0.0009	2.02E-08	0.07
15	rs7171073	57357181	G	A	-0.145	0.08	8.53E-02	-0.005	0.001	2.80E-08	-0.005	0.0009	2.57E-08	0.10
15	rs28422021	57364110	T	C	-0.128	0.08	1.28E-01	-0.005	0.001	3.60E-08	-0.005	0.0009	3.29E-08	0.14
15	rs11856411	57364550	G	A	-0.118	0.08	1.57E-01	-0.005	0.001	3.60E-08	-0.005	0.0009	3.36E-08	0.17
15	rs28541606	57366744	A	G	-0.127	0.08	1.32E-01	-0.005	0.001	3.90E-08	-0.005	0.0009	3.55E-08	0.15
15	rs28490139	57369850	A	G	0.007	0.12	9.54E-01	-0.005	0.001	3.10E-08	-0.005	0.0009	3.11E-08	0.92
15	rs6493900	57371665	T	G	-0.125	0.08	1.38E-01	-0.005	0.001	3.40E-08	-0.005	0.0009	3.13E-08	0.15
15	rs8023736	57376553	G	A	-0.126	0.08	1.34E-01	-0.005	0.001	3.60E-08	-0.005	0.0009	3.28E-08	0.15
15	rs7163267	57377037	G	A	-0.127	0.08	1.30E-01	-0.005	0.001	3.90E-08	-0.005	0.0009	3.54E-08	0.14
15	rs28372003	57383129	A	G	-0.122	0.08	1.46E-01	-0.005	0.001	2.90E-08	-0.005	0.0009	2.64E-08	0.16
15	rs28712697	57383219	G	A	-0.122	0.08	1.46E-01	-0.005	0.001	3.30E-08	-0.005	0.0009	3.01E-08	0.16
15	rs9806170	57389368	G	A	-0.126	0.08	1.39E-01	-0.005	0.001	3.80E-08	-0.005	0.0009	3.50E-08	0.15
15	rs9806199	57390895	T	G	-0.122	0.08	1.48E-01	-0.005	0.001	3.30E-08	-0.005	0.0009	3.05E-08	0.16
15	rs16977243	57394005	C	A	-0.061	0.08	4.70E-01	-0.005	0.001	3.30E-08	-0.005	0.0009	3.20E-08	0.51
15	rs10467927	57401624	G	C	-0.118	0.08	1.59E-01	-0.005	0.001	4.40E-08	-0.005	0.0009	4.01E-08	0.18

15	rs12101455	57404250	G	A	-0.119	0.08	1.56E-01	-0.005	0.001	4.20E-08	-0.005	0.0009	3.83E-08	0.17
15	rs58479554	57404406	C	T	-0.119	0.08	1.56E-01	-0.005	0.001	3.80E-08	-0.005	0.0009	3.54E-08	0.17
15	rs7169508	57411354	A	G	-0.106	0.08	2.12E-01	-0.005	0.001	3.70E-08	-0.005	0.0009	3.48E-08	0.23
15	rs2169467	57414495	G	A	-0.119	0.08	1.58E-01	-0.005	0.001	3.40E-08	-0.005	0.0009	3.09E-08	0.18
15	rs12591430	57416825	C	T	-0.116	0.08	1.69E-01	-0.005	0.001	4.40E-08	-0.005	0.0009	4.04E-08	0.19
15	rs2733333	57435926	A	G	-0.103	0.08	2.23E-01	-0.005	0.001	3.60E-08	-0.005	0.0009	3.34E-08	0.25
15	rs2593252	57449165	A	T	-0.112	0.08	1.85E-01	-0.005	0.001	4.70E-08	-0.005	0.0009	4.38E-08	0.20
15	rs16977270	57453488	G	A	-0.121	0.08	1.53E-01	-0.005	0.001	4.40E-08	-0.005	0.0009	4.08E-08	0.17
15	rs2703614	57459120	G	A	-0.115	0.08	1.74E-01	-0.005	0.001	3.10E-08	-0.005	0.0009	2.89E-08	0.19
15	rs28781690	57466974	A	G	-0.111	0.08	1.90E-01	-0.005	0.001	2.70E-08	-0.005	0.0009	2.47E-08	0.21
15	rs10467928	57480776	G	C	-0.110	0.08	1.90E-01	-0.005	0.001	2.60E-08	-0.005	0.0009	2.40E-08	0.21
15	rs10467929	57480861	G	A	-0.114	0.08	1.79E-01	-0.005	0.001	2.50E-08	-0.005	0.0009	2.27E-08	0.20

Table S2. Meta-analysis results of sexual orientation in Chinese and European population.

SNP	CHR	BP	A1	A2	Chinese Han population			European population			Meta-analysis			
					BETA	SE	P	BETA	SE	P	BETA	SE	P	P_heter
rs2106525	7	115002612	G	C	1.35E-01	8.08E-02	9.33E-02	2.33E-03	4.02E-04	6.60E-09	2.34E-03	4.02E-04	6.24E-09	9.97E-02
rs6970620	7	115005659	A	G	1.44E-01	8.07E-02	7.47E-02	2.33E-03	4.02E-04	7.30E-09	2.33E-03	4.02E-04	6.88E-09	7.89E-02
rs4305836	7	115008063	C	G	1.39E-01	8.08E-02	8.47E-02	2.24E-03	3.99E-04	1.90E-08	2.25E-03	3.99E-04	1.85E-08	9.07E-02
rs4377898	7	115010158	A	T	1.39E-01	8.08E-02	8.47E-02	2.24E-03	3.99E-04	2.00E-08	2.24E-03	3.99E-04	1.89E-08	9.07E-02
rs62474683	7	115020725	A	G	1.39E-01	8.08E-02	8.47E-02	2.23E-03	3.99E-04	2.20E-08	2.24E-03	3.99E-04	2.07E-08	9.07E-02
rs56662562	7	115021945	C	T	1.39E-01	8.08E-02	8.47E-02	2.23E-03	3.99E-04	2.30E-08	2.24E-03	3.99E-04	2.15E-08	9.07E-02
rs10251192	7	115026459	C	T	1.50E-01	8.06E-02	6.25E-02	2.43E-03	4.02E-04	1.60E-09	2.43E-03	4.02E-04	1.49E-09	6.70E-02
rs59369558	7	115029650	T	C	1.40E-01	8.07E-02	8.42E-02	2.24E-03	3.99E-04	2.10E-08	2.24E-03	3.99E-04	2.01E-08	8.85E-02
rs1527743	7	115029890	A	G	1.50E-01	8.06E-02	6.18E-02	2.41E-03	4.02E-04	1.80E-09	2.42E-03	4.02E-04	1.71E-09	6.68E-02
rs17137753	7	115030592	G	A	1.56E-01	8.06E-02	5.32E-02	2.22E-03	3.99E-04	2.60E-08	2.23E-03	3.99E-04	2.42E-08	5.60E-02
rs9641538	7	115031575	G	A	1.50E-01	8.06E-02	6.31E-02	2.41E-03	4.02E-04	1.80E-09	2.42E-03	4.02E-04	1.72E-09	6.69E-02
rs1534373	7	115033079	T	G	1.50E-01	8.06E-02	6.18E-02	2.44E-03	4.02E-04	1.30E-09	2.44E-03	4.02E-04	1.26E-09	6.68E-02
rs12706031	7	115045712	A	C	1.46E-01	8.05E-02	7.02E-02	2.28E-03	4.01E-04	1.40E-08	2.28E-03	4.01E-04	1.29E-08	7.46E-02
rs10239094	7	115053728	G	T	1.55E-01	8.06E-02	5.42E-02	2.28E-03	4.02E-04	1.40E-08	2.28E-03	4.02E-04	1.28E-08	5.75E-02
rs2141233	7	115065555	T	G	1.46E-01	8.07E-02	7.05E-02	2.26E-03	4.02E-04	1.80E-08	2.26E-03	4.02E-04	1.70E-08	7.54E-02
rs1358394	7	115068713	A	G	1.46E-01	8.07E-02	7.03E-02	2.26E-03	4.02E-04	1.90E-08	2.26E-03	4.02E-04	1.83E-08	7.54E-02

Table S3. DEGs participated in serotonin and dopamine metabolic process

	Gene	CTRL	KO	FoldChange	P	Padj	Up/Down
Serotonin metabolic process related DEGs	Arrb2	1712.688268	3590.584422	2.09646115	2.75E-06	0.000429	Up
	Cd300a	707.3927713	1642.150713	2.32141291	0.012764	0.147565	Up
	Fcgr3	925.6778127	2368.145237	2.55828238	0.018537	0.185098	Up
	Pde1b	446.5499399	1145.872407	2.56605657	0.004647	0.080183	Up
Dopamine metabolic process related DEGs	Celsr3	47.47164966	223.5878075	4.70992285	2.79E-03	5.74E-02	Up
	Dlg4	111.0335435	248.8661791	2.24136032	7.77E-03	0.10834	Up
	Entpd1	752.2016074	2377.452867	3.16065912	1.45E-08	5.49E-06	Up
	Gabbr1	431.0747669	1000.427158	2.32077411	7.06E-07	0.000152	Up
	Itgam	2732.99678	6267.118771	2.29313068	0.000211	0.010741	Up
	Nr4a2	6.168390399	27.57304789	4.47005557	0.012353	0.144771	Up
	Palm	172.5745998	446.8747652	2.58945851	0.03625	0.268963	Up
	Ptgs2	73.37283737	880.3395054	11.9981663	0.000325	0.014427	Up
	Vangl2	67.78170931	26.99513678	0.3982658	0.004469	0.077973	Down
	Camk2b	123.3350318	43.00576181	0.34869056	5.41E-05	0.003969	Down
	Fos	1283.51588	8006.937169	6.23828446	0.000344	0.015007	Up
	Gnb2	1394.398188	2827.768691	2.0279492	0.001362	0.036277	Up
	Kif5a	10.13696416	30.64871118	3.02346054	0.020389	0.195679	Up
	Mapk13	41.91749171	160.1113242	3.81967808	0.000372	0.015674	Up
	Ppp2r3d	28.5337414	70.45217333	2.46908291	0.005146	0.085377	Up
	Prkcg	77.26830971	194.6303265	2.5188894	0.019986	0.193322	Up

Table S4. Relative expression level of DEGs

Relative expression level of DEGs												
groups	Arrb2	Cd300a	Fcgr3	Pde1b	App	Dlg4	Entpd1	Gabbr1	Itgam	Palm	Ptgs2	Mapk13
CTRL	1	1	1	1	1	1	1	1	1	1	1	1
KO	1.12123	1.64649	1.74358	1.50136	4.05759	0.8354	2.43103	1.50668	1.91365	1.49915	9.57699	2.76149

Table S5. **Association results for previously-reported regions.**

Chr.	SNP	Position	Allele	MAF		P value
				Homosexual men	Heterosexual controls	
7	rs13244714	147995460	G/A	0.100	0.136	3.43E-03
7	rs2975199	151016648	A/G	0.529	0.480	8.44E-03
7	kgp11476983	151076661	G/C	0.209	0.251	7.73E-03
7	kgp366600	151100144	G/A	0.323	0.370	7.21E-03
7	kgp1078303	151101726	A/C	0.384	0.431	9.06E-03
7	rs7808369	151126822	A/G	0.164	0.130	7.72E-03
7	rs6464215	151955297	A/G	0.083	0.120	1.40E-03
7	rs7787666	152009647	G/A	0.083	0.120	1.40E-03
7	rs13242929	152033193	A/C	0.074	0.120	6.91E-05
7	rs7799662	152036934	G/A	0.077	0.122	1.13E-04
7	rs13240564	152050571	A/G	0.084	0.120	1.88E-03
7	kgp5525439	152053209	G/A	0.084	0.121	1.66E-03
7	kgp7297624	152162319	A/G	0.044	0.075	7.73E-04
7	rs11977276	152719593	A/G	0.374	0.425	5.41E-03
7	rs4355727	154905319	G/A	0.242	0.199	4.09E-03
7	rs6965086	155196066	A/G	0.312	0.268	8.23E-03
7	rs12113582	155974712	C/A	0.159	0.197	7.50E-03
7	rs12113716	155975007	C/A	0.160	0.198	7.95E-03
7	kgp2997033	156372473	C/A	0.149	0.185	8.71E-03
7	rs878715	156383237	G/A	0.454	0.405	7.42E-03
7	rs2178528	158393488	G/A	0.046	0.070	7.26E-03
7	rs10262207	158400183	A/G	0.036	0.057	9.08E-03
7	rs7788516	158410592	C/A	0.034	0.059	1.84E-03
7	rs11983468	158412279	G/A	0.033	0.060	8.99E-04
8	rs608321	29278868	A/G	0.134	0.177	1.93E-03
8	rs7819183	29315425	A/G	0.084	0.056	1.73E-03
8	rs7834608	29319875	G/A	0.181	0.139	1.18E-03
8	rs7824399	32141515	A/G	0.118	0.163	6.01E-04
8	rs901561	32143462	G/A	0.148	0.199	3.99E-04
8	kgp6818187	32429734	A/G	0.194	0.237	4.71E-03
8	kgp5382786	33551744	A/G	0.079	0.054	5.83E-03
8	kgp6266680	33827581	A/C	0.133	0.101	5.29E-03
8	rs1495216	34001156	G/A	0.407	0.359	7.57E-03
8	rs1495219	34011345	G/A	0.377	0.323	1.81E-03
8	rs2729976	34087160	C/A	0.324	0.272	1.80E-03
8	rs13273367	34088496	A/G	0.311	0.264	3.89E-03
8	rs10107864	34901580	A/G	0.423	0.474	5.90E-03

10	rs1006285	119519323	G/A	0.088	0.126	1.28E-03
10	rs3740568	121607024	A/G	0.168	0.132	5.29E-03
10	rs3808959	121652180	G/A	0.174	0.137	4.62E-03
10	kgp314672	121983896	C/A	0.154	0.197	2.43E-03
10	kgp2801665	122005815	C/A	0.156	0.200	2.23E-03
10	rs1999414	122007448	A/C	0.243	0.288	6.34E-03
10	rs7394051	122008026	G/A	0.161	0.199	8.28E-03
10	rs10510063	122020617	G/A	0.159	0.197	8.56E-03
10	kgp4526732	123480747	A/G	0.072	0.048	3.50E-03
10	rs2936874	123490869	C/A	0.364	0.317	6.71E-03
10	rs12257711	123749116	C/A	0.106	0.146	1.31E-03
10	rs10160002	123754074	A/G	0.100	0.140	1.02E-03
10	kgp22043967	123800869	C/A	0.370	0.424	2.65E-03
10	rs2253762	123801422	C/A	0.078	0.107	8.76E-03
10	rs2420993	123857029	A/C	0.214	0.258	5.18E-03
10	rs10736311	124023434	C/A	0.250	0.210	8.62E-03
10	rs2901354	124733038	A/G	0.418	0.372	9.43E-03
10	kgp11765633	124921400	C/A	0.059	0.084	9.78E-03
10	rs1914543	125661410	G/A	0.398	0.449	5.17E-03
10	rs2138923	125662511	A/G	0.398	0.450	4.77E-03
10	kgp1671929	126036215	G/A	0.060	0.086	9.16E-03
10	rs11244450	126036778	A/G	0.082	0.116	2.15E-03
10	rs17619406	126090430	G/A	0.057	0.082	7.58E-03
10	kgp437698	127904435	A/G	0.123	0.160	5.14E-03
10	kgp5489536	128164803	G/A	0.438	0.389	6.85E-03
10	rs10443996	129394566	C/A	0.073	0.110	8.46E-04
10	rs7901816	129401906	A/G	0.081	0.117	1.32E-03
10	kgp4337472	130303915	A/G	0.037	0.061	5.22E-03
10	rs12217588	130354468	A/G	0.418	0.366	3.37E-03
10	kgp9231132	130510150	A/G	0.087	0.117	9.84E-03
10	rs672996	132212976	G/A	0.183	0.146	5.85E-03
10	rs165517	132220631	A/G	0.187	0.148	3.57E-03
10	kgp21568210	133031628	A/G	0.054	0.080	6.11E-03
10	kgp1630797	133031768	G/A	0.057	0.083	6.81E-03
10	rs12414883	133120100	A/C	0.112	0.081	4.20E-03
10	kgp1344882	133836794	T/A	0.198	0.160	6.17E-03
10	rs12769959	134579026	C/A	0.107	0.139	9.24E-03
23	rs4843993	147452494	G/A	0.189	0.152	6.94E-03
23	rs5936343	147455455	A/G	0.187	0.152	9.95E-03
23	rs2335517	147469249	A/G	0.182	0.147	7.53E-03

23	rs5980519	147516697	G/A	0.170	0.129	1.30E-03
23	rs729815	147521049	A/G	0.169	0.124	3.67E-04
23	rs5936373	147528439	A/G	0.136	0.092	8.74E-05
23	rs5980526	147533488	A/C	0.144	0.094	1.47E-05
23	rs3762238	147581166	A/G	0.065	0.044	8.01E-03
23	rs5980538	147581470	G/A	0.065	0.044	8.45E-03
23	kgp22795021	148290849	A/G	0.092	0.067	9.15E-03
23	rs7391189c	149219447	C/A	0.119	0.192	1.42E-07
23	rs5969969	149498146	G/A	0.265	0.320	1.30E-03
23	rs548975	149627126	G/A	0.136	0.103	4.31E-03
23	rs579854c	149628600	C/A	0.094	0.049	3.53E-07
23	rs593088	149628916	A/G	0.138	0.103	2.45E-03
23	rs477252	149648283	A/G	0.321	0.270	2.44E-03
23	rs2266836	149659739	A/G	0.489	0.441	7.76E-03
23	rs685809	149663850	A/G	0.493	0.431	5.96E-04
23	rs5969978	150114170	G/A	0.403	0.457	3.25E-03
23	rs4828787	150164884	G/A	0.108	0.156	1.75E-04
23	kgp22783066	150259920	G/A	0.273	0.225	2.25E-03
23	rs6627851	150266861	G/A	0.347	0.301	6.00E-03
23	rs5970539	150280253	G/A	0.036	0.079	2.92E-06
23	rs552922	150281061	G/A	0.031	0.061	1.92E-04
23	rs481878	150350784	G/A	0.286	0.335	4.56E-03
23	rs2734239	150462517	G/A	0.321	0.384	3.78E-04
23	rs4828661	151030048	G/A	0.173	0.128	4.72E-04
23	rs1109576	151158776	G/A	0.395	0.329	1.75E-04
23	rs2471365	151245139	G/A	0.091	0.123	6.28E-03
23	kgp22749260	151369277	A/C	0.338	0.394	1.61E-03
23	rs4898428	152876697	G/A	0.497	0.449	9.07E-03
23	rs5945327	152900090	A/G	0.500	0.432	1.95E-04
23	rs3761536	152906292	T/A	0.431	0.379	4.69E-03
23	rs3761534	152907551	G/A	0.504	0.432	9.05E-05
23	rs633	153274228	A/G	0.173	0.222	8.97E-04
23	rs3027898	153275890	A/C	0.175	0.222	1.52E-03
23	rs1059702	153284192	G/A	0.198	0.241	5.62E-03
23	rs17435	153311980	A/T	0.190	0.231	6.67E-03
23	rs1624766	153317154	A/G	0.175	0.225	8.14E-04
23	rs1734787	153325446	A/C	0.188	0.240	7.99E-04
23	rs1734791	153330920	T/A	0.192	0.242	1.18E-03
23	rs2239464	153348431	G/A	0.177	0.231	4.01E-04
23	rs7054137	153752240	G/A	0.035	0.061	1.24E-03

23	kgp22835957	153755989	A/G	0.036	0.064	1.27E-03
23	kgp22823406	154512663	G/A	0.037	0.061	2.79E-03
23	kgp22799002	154921994	A/G	0.037	0.062	2.18E-03
23	kgp22747224	154987147	G/A	0.503	0.436	2.38E-04
23	kgp22752877	155097176	A/T	0.391	0.449	1.63E-03
23	kgp22769142	155119648	G/C	0.310	0.369	6.97E-04
23	kgp22820064	155158490	A/G	0.313	0.367	2.26E-03
23	kgp22780361	155158531	G/A	0.313	0.366	2.48E-03
23	kgp22800631	155167945	G/A	0.317	0.369	3.25E-03

Table S6. The sequences of primer list used in the study

name	sequence
FMR1NB-sens	TAAGTCCCAGGAGGTTCTGGACTG
FMR1NB-anti	TACCTCACACTTCCCAAGTCCCT

Table S7. The primer sequences of DEGs

gene	forward primer	reverse primer
Arrb2	CGTCCATGTCACCAACAAT	TTGTTCTAGCTGAGCCACG
Cd300a	TGGGAATTGACATGGTTCGT	TTCTTCCTGGACTGACACTAC
Fcgr3	CAAACTACTGAACAGGATCTCG	TGTAGTGATGATACCTCACGG
Pde1b	CCACCTGGACCAAGTACA	ACATCTGGTTGGTGATGC
Dlg4	GACCGATGACATTGGCTT	TGCGTCACCGTCTCATAG
Entpd1	AAGCCAAGTGTCCTGAATC	TAGCACATGACCACAGACG
Gabbr1	CCCAACCTGAACAATCTGAC	CTGGCTTCTCCCTATGTGGTAA
Itgam	AGGAGTGTGTTTGCATGT	GTCCTTGGTGTTCTTGCG
Palm	GAGATGGAGGTCCTGGCAA	CAATCTCTGCCTGCTTCCTA
Ptgs2	CATGGGTGTGAAGGGAAATAAG	ATGCAAACATCATATTTGAGCC
Mapk13	ACCAGATGCTCAAAGGTCTAA	TCTTCAGCTCACAGTCTTCATT
GAPGH	GCAAGGACACTGAGCAAGA	GGATGGAAATTGTGAGGGAG
